# Ailanthone targets p23 to overcome MDV3100 resistance in castration-resistant prostate cancer

**DOI:** 10.1038/ncomms13122

**Published:** 2016-12-13

**Authors:** Yundong He, Shihong Peng, Jinhua Wang, Huang Chen, Xiaonan Cong, Ang Chen, Meichun Hu, Min Qin, Haigang Wu, Shuman Gao, Liguo Wang, Xin Wang, Zhengfang Yi, Mingyao Liu

**Affiliations:** 1 East China Normal University and Shanghai Fengxian District Central Hospital Joint Center for Translational Medicine, Shanghai Key Laboratory of Regulatory Biology, Institute of Biomedical Sciences and School of Life Sciences, East China Normal University, Shanghai 200241, China; 2 Division of Biomedical Statistics and Informatics, Mayo Clinic College of Medicine, Rochester, Minnesota 55905, USA; 3 Institute of Biosciences and Technology, Department of Molecular and Cellular Medicine, Texas A&M University Health Science Center, Houston, Texas 77030, USA

## Abstract

Androgen receptor (AR) antagonist MDV3100 is the first therapeutic approach in treating castration-resistant prostate cancer (CRPC), but tumours frequently become drug resistant via multiple mechanisms including AR amplification and mutation. Here we identify the small molecule Ailanthone (AIL) as a potent inhibitor of both full-length AR (AR-FL) and constitutively active truncated AR splice variants (AR-Vs). AIL binds to the co-chaperone protein p23 and prevents AR's interaction with HSP90, thus resulting in the disruption of the AR-chaperone complex followed by ubiquitin/proteasome-mediated degradation of AR as well as other p23 clients including AKT and Cdk4, and downregulates AR and its target genes in PCa cell lines and orthotopic animal tumours. In addition, AIL blocks tumour growth and metastasis of CRPC. Finally, AIL possesses favourable drug-like properties such as good bioavailability, high solubility, lack of CYP inhibition and low hepatotoxicity. In general, AIL is a potential candidate for the treatment of CRPC.

## Abstract (web summary)

Prostate cancers often become castration resistant due to alternative expression of androgen receptor (AR) splice variants. Here, the authors screened a library of natural compounds and identified Ailanthone as a potent inhibitor of AR through its binding to the co-chaperone protein p23 that, by preventing AR interaction with HSP90, results in ubiquitin/proteasome-mediated degradation of the receptor.

Prostate cancer (PCa) is the most common male cancer in many industrialized countries^1^,^2^. PCa initially depends on androgen receptor (AR) signalling for growth and survival. Androgen ablation therapy causes a temporary reduction in PCa tumour burden, but the tumour eventually develops into castration-resistant prostate cancer (CRPC) with the ability to grow again in the absence of androgens^3^. Mechanisms of CRPC progression include AR amplification and overexpression^4^,^5^, AR gene rearrangement promoting synthesis of constitutively active truncated AR splice variants (AR-Vs)^6^ and induction of intracrine androgen metabolic enzymes^3^,^7^. The canonical human AR has 919 amino acids with a mass of 110 kDa, composed of four structurally and functionally distinct domains including the N-terminal domain (amino acids 1–537), DNA-binding domain (amino acids 537–625), hinge region (amino acids 625–669) and ligand-binding domain (LBD, amino acids 669–919)^8^. When activated by endogenous androgens, AR translocates into the nucleus, associates with coregulatory factors and binds to specific genomic DNA sequences in the regulatory regions of AR target genes^9^. Previous clinical research showed that targeting AR was a valid therapeutic strategy for CRPC^10^. Indeed, recent clinical trials have shown that the AR antagonist MDV3100 (MDV)^11^ and abiraterone, an inhibitor targeting androgen synthesis^12^, are effective against CRPC. However, recent studies have reported that AR-Vs which lack the LBD are resistant to anti-androgen therapy including MDV and abiraterone^13^,^14^,^15^,^16^,^17^. Since the major AR-Vs identified to date have an intact N-terminal domain and DNA-binding domain, they display constitutive activity, which underlies the persistent AR signalling in CRPC expressing these variants^6^,^18^,^19^,^20^. Collectively, both ligand-dependent full-length AR (AR-FL) and AR-Vs mediate distinct transcriptional programs in CRPC^21^,^22^,^23^, but AR inhibitors currently in clinical use all target the LBD, and thus would not overcome cancer cell resistance driven by constitutively active AR-Vs.

AR is maintained in a ligand-binding competent state through its interaction with the foldosome, a protein complex consisting of the chaperones HSP40, HSP70 and HSP90 together with the co-chaperones HOP, p23 and the immunophilins FKBP51/52 and BAG-1 (ref. 24). Intriguingly, some inhibitors of HSP90 such as AT13387 decrease the expression of several HSP90 client proteins including wild-type AR and AR–V7 (an AR splice variant), and also disrupt nuclear localization of the AR. A phase I/II clinical trial of AT13387 alone or in combination with abiraterone acetate in patients with mCRPC is in progress^25^. Other HSP90 inhibitors that target the HSP90 N terminus including NVP-HSP990 and PF-04929113 have activity in preclinical studies^26^,^27^. The co-chaperone p23 is overexpressed in multiple types of cancer, and protects cancer cells from HSP90 inhibitors^28^. p23 overexpression is induced on treatment with either androgens or anti-androgens and facilitates PCa cell motility; p23 knockdown inhibits the invasiveness of the PCa cell line LNCaP, suggesting an important role of p23 in PCa metastasis independent of its role as an HSP90 co-chaperone^29^. The expression of p23 increases AR protein level, AR ligand-binding activity and AR's target promoter-binding activity; most importantly, p23 functions to promote AR activity in an HSP90-independent mechanism involving the direct binding to AR^30^. p23 is also associated with an increased resistance to etoposide and doxorubicin in breast cancer cells^31^ along with elevated expression of a subset of estrogen-responsive genes^32^. p23 overexpression correlates with poor prognosis for breast cancer patients, implicating p23's role in breast cancer progression in addition to PCa, supporting the utility of p23 as a potential therapeutic target for cancer therapy.

To identify compounds that block the transcriptional activities of both ligand-dependent AR-FL and constitutively active AR-Vs, we used the MMTV-luciferase (MMTV-luc) reporter system containing AR-binding elements^33^ to screen ∼100 compounds from a library of natural compounds (including about 1,000 natural compounds extracted from Traditional Chinese Medicine) (Supplementary Tables 4 and 5) and identified a small-molecule compound termed Ailanthone (AIL), which is a natural compound extracted from the whole seedlings of *Ailanthus altissima* (Simaroubaceae) that has antimalarial and antitumour activities^34^,^35^. In this study, we find that AIL potently reduce the transcriptional activities of both AR-FL and AR-Vs. In addition, AIL decrease the protein levels of not only AR-FL but also constitutively active AR-Vs, resulting in cell growth inhibition as well as suppression of MDV3100-resistant CRPC metastasis, by binding to p23 protein. Furthermore, we evaluate the drug-like properties of AIL including solubility, pharmacokinetics, bioavailability, cytochrome P450 (CYP) inhibition and toxicity. Overall, our findings provide the first evidence that AIL is a promising lead compound against CRPC and is suitable for further pharmaceutical development.

## Results

### AIL suppresses the activities of AR-FL and AR-Vs

To identify compounds that inhibit the transcriptional activities of both AR-FL and constitutively active AR-Vs, we used a luciferase reporter assay to screen about 100 compounds from a library of natural compounds. 22RV1 PCa cells were either stimulated with androgen dihydrotestosterone (DHT) to activate AR-FL or transfected with AR_1-651_ to introduce the splice variant of AR lacking the LBD. After incubation with these natural compounds for 12 h, the transfected cells were collected and AR transcriptional activity was detected by dual luciferase assay (Supplementary Tables 4 and 5). We identified the small-molecule AIL that potently reduced the transcriptional activities of both AR-FL and AR-Vs. The physicochemical properties of AIL are listed in Supplementary Table 1.

**Figure 1:**
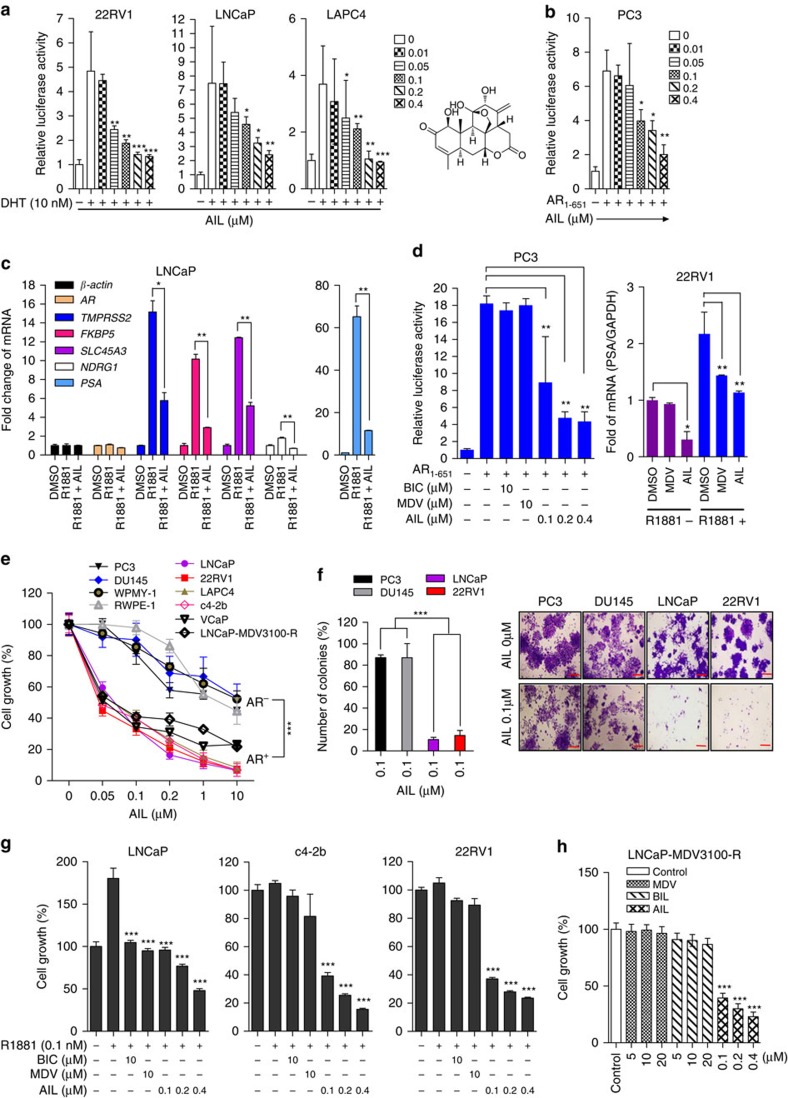
Inhibitory effects of AIL on AR activity and PCa cell proliferation. (**a**,**b**) PCa cells were treated with different concentrations of AIL for 12 h and luciferase activities were measured. MMTV-luc reporter was stimulated by DHT (**a**) or exogenous AR_1–651_ (**b**). (**c**) LNCaP cells were cultured in 5% c-FBS for 5 days and treated with 10 nM R1881 alone or 0.2 μM AIL with 10 nM R1881 for 12 h. The gene expression was measured by quantitative-PCR. (**d**) PC3 cells were transfected with AR_1–651_, MMTV-luc, Renilla-luc and treated with indicated concentrations of bicalutamide (BIC), MDV3100 (MDV) and AIL. After 12 h, the MMTV-luc activities were detected (left panel). 22RV1 cells were stimulated with or without 10 nM R1881 and treated with 10 μM MDV or 0.2 μM AIL. After 12 h, total RNA was extracted and *PSA* mRNA was measured by quantitative-PCR (right panel). (**e**,**f**) The AR negative cell lines PC3 and DU145 (AR^−^), normal prostate cell lines RWPE-1 and WPMY-1 (N), and AR positive cell lines LNCaP, 22RV1, LAPC4 c4-2b,VcaP and LNCaP-MDV3100-R (AR^+^) were treated with different concentrations of AIL for 48 h. Cell proliferation was detected with the SRB assay (**e**). AR negative cell lines PC3 and DU145, and positive cell lines LNCaP, 22RV1 were treated with 0 or 0.1 μM AIL for 7 days, and the cell colonies were counted (**f**). Data were expressed as mean±s.d. of three independent assays; two-way ANOVA followed by Bonferroni multiple comparison test; ***P<0.001. Scale bar, 200 μm. (**g**,**h**) Androgen-starved LNCaP, c4-2b and 22RV1 cells were treated with 10 μM BIC, 10 μM MDV and 0.1, 0.2, 0.4 μM AIL together with 0.1 nM R1881 stimulation for 96 h (**g**). MDV3100-resistant LNCaP cells were treated with the indicated concentrations of BIC, MDV or AIL for 72 h (**h**). Cell growth was determined by SRB assay. In **a**–**d**,**g** and **h**, data were expressed as mean±s.d. of three independent assays; Student's *t*-tests were performed; *P<0.05, **P<0.01, ***P<0.001.

To further test the bioactivity of AIL (structure shown in Fig. 1a, right panel), luciferase reporter assays were performed in several PCa cell lines including LNCaP, c4-2b, 22RV1 and AR-transfected PC3 cells. As shown in Fig. 1a,b, AIL dose-dependently inhibited the DHT-induced transcriptional activities of AR and constitutively active truncated AR_1–651_ at low concentrations (AR-FL IC_50_=69 nM, 95% confidence interval=53–89 nM; AR_1–651_ IC_50_=309 nM, 95% confidence interval=236–687 nM in 22RV1 cells). The AIL-mediated repression of AR activity was also observed in PC3 cells co-transfected with the AR expression vector plasmid and reporters (Supplementary Fig. 7g).

To examine whether AIL had an effect on AR-dependent endogenous gene expression, the levels of mRNA transcripts for numerous well-characterized AR-regulated genes were measured in LNCaP cells. As shown in Fig. 1c, AIL decreased the androgen-dependent induction of endogenous *PSA*, *TMPRSS2*, *FKBP5*, *SLC45A3* and *NDRG1* mRNA expression. Since AR-Vs lacking the LBD are resistant to AR antagonists, we next investigated whether AIL blocked its constitutive and androgen-independent AR activity. As shown in Fig. 1d, the constitutively active truncated AR_1–651_ lacking the LBD was resistant to the AR antagonists bicalutamide (BIC) and MDV, but its transcriptional activity was also blocked by AIL in a dose-dependent manner (Fig. 1d, left panel). Similarly, in 22RV1 cells that naturally express AR-Vs, although MDV decreased the level of the AR target gene PSA in the presence of the synthetic androgen methyltrienolone (R1881), it had no effect in the absence of R1881. However, AIL downregulated PSA not only in the presence but also in the absence of R1881 (Fig. 1d, right panel). Taken together, AIL inhibited the activity of both the androgen inducible AR-FL and the constitutively active truncated AR lacking the LBD.

### AIL inhibits the proliferation of PCa cells

We examined whether AIL affected the proliferation of AR positive PCa cells. Using the sulforhodamine B colorimetric (SRB) assay, we confirmed that AIL potently inhibited the growth of several PCa cell lines including LNCaP, c4-2b, 22RV1 and LAPC4 (Supplementary Fig. 1a). In addition, AIL induced G1-phase arrest instead of apoptosis (Supplementary Fig. 8a,b; Supplementary Methods). Interestingly, AIL more potently inhibited the growth of AR positive prostate cancer cells than either AR negative tumour cell lines or normal prostate cell lines (Fig. 1e, Supplementary Figs 1b and 2). In the colony formation experiments, AR positive cells were also more sensitive to AIL (Fig. 1f). Moreover, in the transwell chamber migration assay, AIL suppressed AR-positive LNCaP cell migration more effectively than that of AR-negative PC3 cells (Supplementary Fig. 1c).

To examine whether AIL could overcome the resistance to androgen antagonist therapy, LNCaP, c4-2b and 22RV1 cells were tested using the SRB assay (Fig. 1g). Although C4-2b and 22Rv1 cells may be androgen-insensitive, these assays were performed in the presence of R1881. In androgen sensitive LNCaP cells, the well-known AR antagonists BIC and MDV effectively blocked cell growth as well as AIL (Fig. 1g). However, in the androgen-insensitive c4-2b line and the CRPC cell line 22RV1, 10 μM BIC and 10 μM MDV could not significantly inhibit cell growth, but 0.1 μM AIL remarkably inhibited growth (Fig. 1g). Furthermore, LNCaP-MDV3100-R cells (a MDV3100-resistant LNCaP cell subline that was chronically cultured in the presence of MDV and is characterized in Supplementary Fig. 12) were totally resistant to BIC and MDV at a high concentration (20 μM), but 0.1 μM AIL treatment still significantly induced cell growth arrest (Fig. 1h). Collectively, AIL inhibited both androgen-dependent and androgen-independent PCa cell growth and overcame resistance to AR antagonist therapy.

### AIL blocks tumour growth and metastasis of CRPC

We evaluated the efficacy of AIL *in vivo* by treating 22RV1 xenografts in male BALB/c nude mice with AIL for 35 days. Administration of 1 and 3 mg kg^−1^ per day AIL significantly inhibited the increase of tumour volume in 22RV1 xenografts (Supplementary Fig. 3). AIL did not significantly affect the body weight of mice and did not show apparent toxicity as determined by pathological review of sections of lungs, heart, liver, spleen and kidneys collected from mice receiving AIL (Supplementary Fig. 5a,b). In addition, treatment with AIL decreased the weight of seminal vesicle of the mice (Supplementary Fig. 5c), indicating that AIL blocked AR signalling in the mice *in vivo*. Therefore, we selected the dose of 2 mg kg^−1^ per day AIL for further experiments in animals.

**Figure 2:**
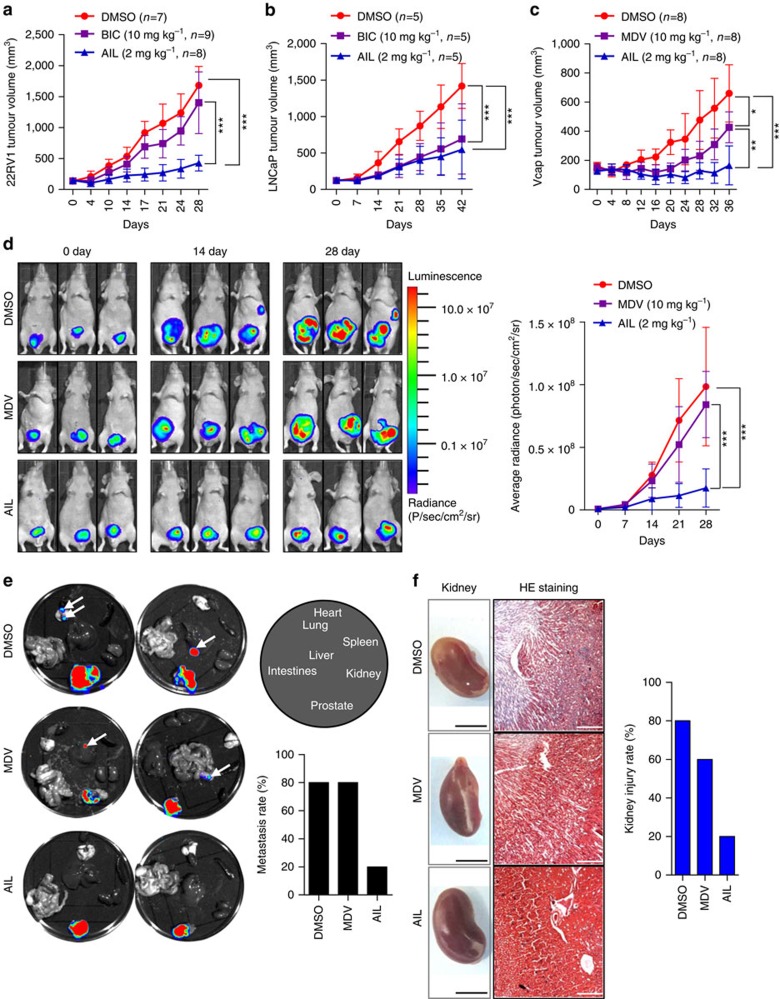
Therapeutic effects of AIL on castration-resistant xenografts. (**a**–**c**) 22RV1 (**a**), LNCaP (**b**) and VCaP (**c**) cells suspended in 0.1 ml PBS (22RV1) or matrigel (LNCaP, VCaP) were injected into the right flank of BALB/c-nude mice. Androgen containing blocks were subcutaneously inserted into each mouse in the LNCaP and VCaP xenograft models. After the volume of tumour nodules reached about 100 mm^3^, the mice were randomly assigned to the indicated groups and respectively i.p. injected with vehicle control, AIL, BIC or MDV as indicated. The control group was injected with DMSO. Tumour volume and the mouse body weight were measured twice per week. (**d**) Male BALB/c-nude mice were anaesthetized, and the dorsolateral prostate was injected with 22RV1-luc cells in matrigel. After a week, mice were castrated and treated i.p. with DMSO, 10 mg kg^−1^ MDV or 2 mg kg^−1^ AIL once per day. Tumours were imaged every week to determine local tumour growth and evidence of tumour cell dissemination. Representative images of three mice per group were illustrated (*n*=5). (**e**) After 28 days, mice were killed and local tumours and viscera of the mice were imaged to determine tumour growth and evidence of tumour cell dissemination. Representative images of the dishes are shown, and the number of mice which had metastatic tumours was counted (*n*=5). (**f**) The mouse kidneys from the same experiment as **d** were histopathologically evaluated. The number of mice that had kidney injury was counted (*n*=5). Scale bars: the black scale bar is 0.5 cm (left) and the white scale bar is 100 μm (right). Data represent the mean±s.d. *P<0.05, **P<0.01, ***P<0.001 by one-way ANOVA followed by Bonferroni multiple comparison test.

We also compared the efficiency of AIL with the well-known AR-antagonist BIC in both LNCaP and 22RV1 xenografts. For androgen-sensitive LNCaP cells, treatment with either 10 mg kg^−1^ per day BIC or 2 mg kg^−1^ per day AIL significantly reduced the tumour volume (Figs 2b and 4b). In contrast, the CRPC 22RV1 xenografts were resistant to BIC administration, but AIL strongly inhibited tumour growth (Figs 2a and 4a). Furthermore, we compared the efficiency of AIL with the next generation AR-antagonist MDV in another cell line, VCaP, which expresses AR-Vs but is still sensitive to androgen. As shown in Fig. 2c and Supplementary Fig. 4c, VCaP xenografts were more sensitive to AIL compared with MDV3100, although VCaP xenografts still responded to MDV3100.

To more closely mimic human disease, we further evaluated whether AIL regressed CRPC *in vivo.* Castrated mice bearing 22RV1-luc orthotopic xenografts were treated with AIL. As shown in Fig. 2d and Supplementary Fig. 6a, AIL suppressed the 22RV1 orthotopic xenografts in castrated mice, whereas these CRPC xenografts were resistant to MDV. AIL administration reduced the tumour volume by 82% (95% confidence interval=70–95%), whereas MDV treatment reduced the tumour volume by only 15% (95% confidence interval=0–36%). In addition, AIL inhibited tumour metastasis and reduced kidney injury in this CRPC model. Eighty percent of control mice but only 20% of AIL-treated mice had obvious metastasis (Fig. 2e and Supplementary Fig. 6b) and kidney injury (Fig. 2f). In summary, AIL not only inhibited the tumour growth and metastasis of MDV-resistant 22RV1 cells, but also reduced kidney injury and metastases in orthotopic xenografts.

### AIL downregulates AR protein level *in vitro* and *in vivo*

**Figure 3:**
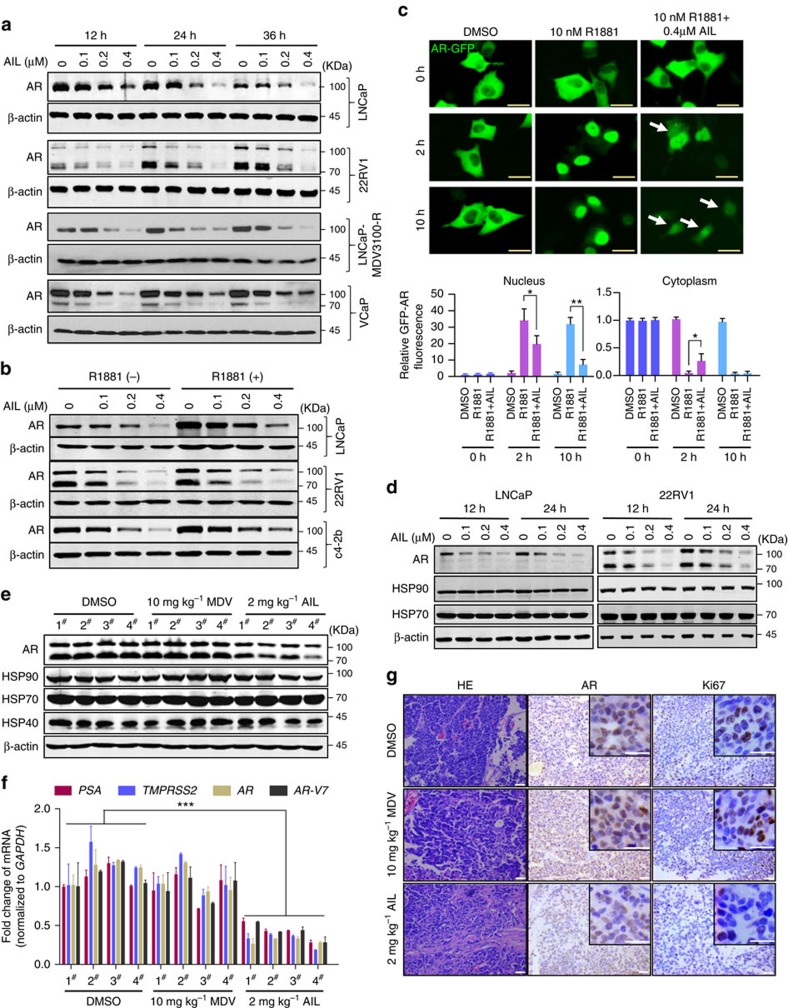
Downregulation of AR protein levels *in vitro* and *in vivo* by AIL. (**a**) LNCaP, 22RV1, LNCaP-MDV3100-R and VCaP cells were treated with indicated concentrations of AIL for 12, 24 and 36 h. Then cells were lysed and AR protein level was measured by western blotting analysis. (**b**) LNCaP, 22RV1 and c4-2b cells were treated for 12 h with indicated concentrations of AIL with or without R1881 and the AR protein level was measured by western blotting analysis. (**c**) AR null PC3 cells were transfected with AR-GFP in serum-free conditions for 24 h and treated with DMSO (control), R1881 (10 nM) or combined AIL (0.4 μM) and R1881 (10 nM). AR-GFP images were taken at 2 or 10 h after treatment. Five pictures were randomly selected and the GFP-AR fluorescence in cytoplasm and nucleus was quantitated using Image-Pro Plus 4.5 software (Media Cybernetics, Silver Spring, USA). The arrows indicate the location of AR-GFP. Data were expressed as mean±s.d.; Student's *t*-tests were performed; *P<0.05, **P<0.01. Scale bars, 10 μm. (**d**) LNCaP and 22RV1 cells were treated with the indicated concentrations of AIL for 12 and 24 h. Cells were lysed and AR, HSP90 and HSP70 protein levels were measured by western blotting analysis. (**e**) Four representative tumour samples per group were lysed. AR, HSP90, HSP70 and HSP40 protein levels were measured by western blotting analysis. (**f**) The mRNA levels of *PSA*, *TMPRSS2*, total *AR* and *AR-V7* were measured by quantitative-PCR and normalized to *GAPDH*. The sequences of quantitative-PCR primers were listed in Supplementary Table 2. Data were expressed as mean±s.d.; two-way ANOVA followed by Bonferroni multiple comparison test were performed; ***P<0.001. (**g**) Photographs of xenografts treated i.p. with DMSO (control group), 10 mg kg^−1^ MDV and 2 mg kg^−1^ AIL with corresponding IHC for AR and Ki67. Scale bars, 20 μm.

To investigate the mechanism of AR transcriptional activity inhibition by AIL, we first determined the AR protein level after AIL treatment in PCa cell lines. AIL potently reduced AR protein expression in a dose-dependent manner in LNCaP, 22RV1, LNCaP-MDV3100-R, and VCaP cell lines (Fig. 3a). In AR positive PCa cell lines, AR was more stable and had a higher basal level in the presence of synthetic androgen R1881; we observed that AIL reduced the AR protein level both in the absence and in the presence of R1881 (Fig. 3b and Supplementary Fig. 7a). Notably, AIL downregulated the truncated splice variants of AR (Supplementary Fig. 7b,c) which were continually active and resistant to AR antagonist therapy. Indeed, knockdown of AR-Vs decreased the proliferation of VCaP and 22RV1 cells which have high expression of AR-Vs (Supplementary Fig. 10c,d). Furthermore, we examined whether AIL prevented AR nuclear translocation by transfecting an AR-GFP fusion protein into PC3 cells. As expected, the nuclear translocation of AR-GFP induced by R1881 was decreased by AIL in PC3 cells (Fig. 3c and Supplementary Fig. 7d). The HSP90 complex plays a major role in stabilizing unliganded AR^24^. Therefore, we examined whether AIL affected the members of the HSP90 complex. Unexpectedly, AIL did not downregulate the AR molecular chaperones HSP90 and HSP70 in PCa cells (Fig. 3d). We further confirmed this phenomenon in the AIL-treated 22RV1 orthotopic xenografts. As demonstrated in Fig. 3e,f, AIL reduced the expression of AR protein and its target genes but had no effect on the AR molecular chaperones HSP90, HSP70 and HSP40 *in vivo*. AR downregulation and proliferation inhibition by AIL treatment in 22RV1 orthotopic xenografts were also confirmed by immunohistochemistry (Fig. 3g). In addition, in an *in vivo* assay, treatment with AIL decreased the mRNA level of the AR-splice variant AR-V7 as well as total AR (Fig. 3f), which might be caused by a secondary effect of long-term AIL treatment.

### Induction of AR degradation by AIL

**Figure 4:**
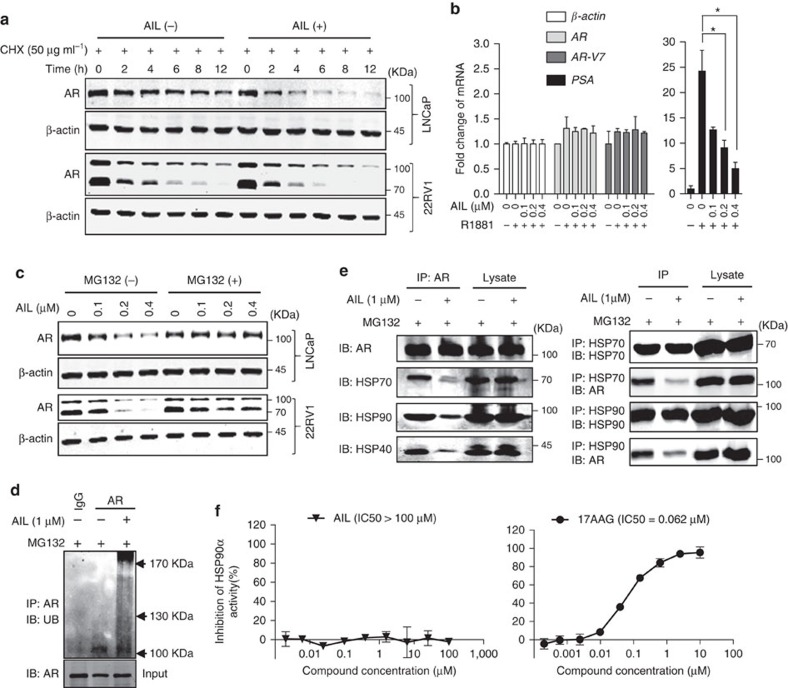
Reduction of AR protein stability by AIL. (**a**) LNCaP and 22RV1 cells were treated with cycloheximide (CHX) with or without AIL for various lengths of time. AR protein level was measured by western blotting analysis. (**b**) LNCaP cells were cultured in c-FBS for 5 days and treated with AIL in the absence or presence of 10 nM R1881 for 12 h. Total RNA was extracted and quantitative-PCR was performed. The *AR-V7* mRNA level was detected in 22RV1 cells that were treated with the indicated concentrations of AIL for 12 h. The expression of *AR*, *AR-V7* and *PSA* was normalized to *β-actin* expression. Data were expressed as mean±s.d. of three independent assays; Student's *t*-tests were performed; *P<0.05. (**c**) LNCaP and 22RV1 cells were treated with various concentrations of AIL with or without MG132 (10 μM) and AR protein level was measured by western blotting analysis. (**d**) Immunoprecipitation (IP) was done using anti-AR and immunoblotting performed with an anti-ubiquitin antibody. Input: immunoblot of lysates probed with AR antibody. (**e**) 22RV1 cells were treated with or without AIL in the presence of MG132. IP was done using anti-AR, anti-HSP90 and anti-HSP70 antibodies and immunoblotting (IB) was done with anti-AR, anti-HSP90, anti-HSP70 and anti-HSP40 antibodies. (**f**) HSP90α activity was measured by fluorescence polarization binding assay using FITC-geldanamycin in the presence of AIL or 17-AAG (positive control).

To investigate why AIL reduced the expression of AR protein but not its molecular chaperones, we tested the effect of AIL on AR protein stability. Surprisingly, AR protein stability was significantly reduced under AIL treatment (Fig. 4a). However, there was no significant effect on AR and AR-V7 mRNA when treated with the same concentration of AIL, although the PSA mRNA level was decreased (Fig. 4b). To test whether AIL induced AR degradation through the proteasome pathway, we treated cells with the proteasome inhibitor MG-132, which resulted in a marked suppression of AIL-induced AR depletion (Fig. 4c). More importantly, treatment with AIL induced ubiquitination of AR (Fig. 4d). Interestingly, while AIL treatment decreased AR, AKT as well as Cdk4 protein levels, it did not influence their chaperones HSP90, HSP70 and HSP40 which were all essential for the HSP90-HSP70 chaperone complex (Supplementary Fig. 7f). To further illustrate this mechanism, we performed co-immunoprecipitation and observed that AIL prevented the interaction of AR with HSP90 and HSP70 as well as HSP40 (Fig. 4e). Together with the decreased protein stability, these data suggested that AIL might induce AR degradation by disrupting the interaction of AR with its chaperones HSP90 and HSP70, resulting in AR ubiquitination and degradation. In addition, AIL treatment led to AKT and Cdk4 downregulation, potentially driving the decreased proliferation in AIL-treated cells.

Given that AIL disrupted the interaction of AR with its chaperone HSP90, we then tested whether AIL inhibited HSP90 activity. Using the geldanamycin-FITC fluorescence polarization assay, we found that AIL did not inhibit HSP90 activity (Fig. 4f). We also observed that 17-AAG induced upregulation of HSP90 and HSP70 protein but AIL did not influence them (Supplementary Fig. 7e), suggesting that AIL, unlike 17-AAG, was not an HSP90 inhibitor.

### Interaction of AIL with p23 *in vitro*

**Figure 5:**
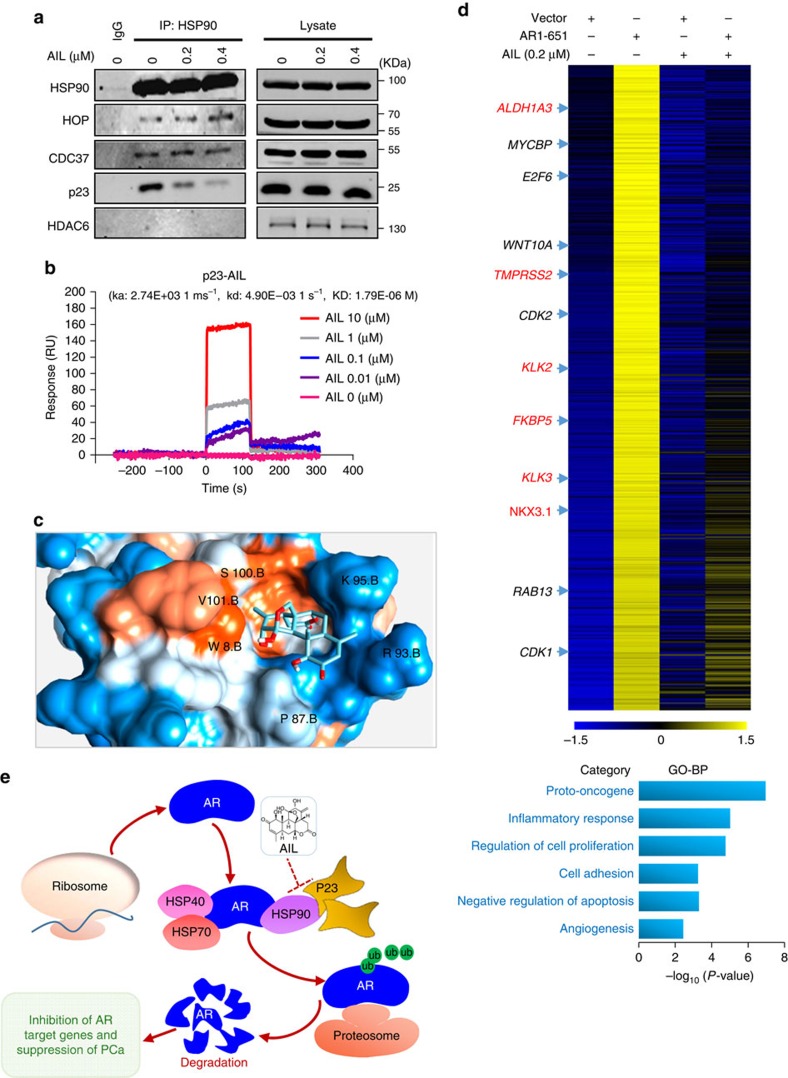
Interaction of AIL with p23 protein *in vitro*. (**a**) 22RV1 cells were treated with or without 1 μM AIL for 12 h. Anti-HSP90 IP was done and co-immunoprecipitated proteins were detected using indicated antibodies. (**b**) The interaction between p23 protein and AIL was measured by ProteOn XPR36. (**c**) Mapping AIL binding site on p23. (**d**) Top: expression level heatmap (log2 based) of AR_1–651_ induced genes which were inhibited by AIL. LNCaP cells were treated as described in Methods and the RNA samples of the indicated groups were sent for RNA-seq. First gene expression values (RPKM) were normalized (z-score transformed) across samples. Then the K-means clustering method was used to portion all genes into the clusters with Pearson correlation as the metric of distance. In the heat map, yellow means ‘higher' expression and blue means ‘lower' expression. Refseq IDs were converted into gene symbols listed in Supplementary Data 1. Bottom: Gene Ontology (GO) analysis of the AR_1–651_ target genes which inhibited by AIL. (**e**) Schematic illustrating the mechanism of downregulating AR protein level by AIL. When treating with AIL, the interaction of p23 and HSP90 is prevented and the interaction between AR and the molecular-chaperones is decreased, causing ubiquitination of AR. Then, AR is degraded by the proteasome, which reduces the expression of AR target genes and inhibits PCa growth and metastasis.

Foldosome complex assembly occurs through a series of steps, beginning with HSP40 and HSP70 binding to AR, followed by HSP90 and HOP, and then succeeded by ATP-dependent binding of p23, FKBP51 and FKBP52 which displace HSP40, HSP70, and HOP^24^. Additional foldosome proteins include cdc37 and HDAC6 (36). Accordingly, we next determined whether AIL disturbed the interaction between these proteins and HSP90. AIL obviously prevented the interaction between p23 and HSP90, but had no significant influence on the interaction of other proteins with HSP90 (Fig. 5a). By Biacore assay, we confirmed that there was no interaction between AIL and HSP90 (Supplementary Fig. 9a; Supplementary Methods). However, AIL interacted directly with p23 (*K*_D_=1.79 × 10^−6^ M) (Fig. 5b). Celastrol (CEL) was used as a positive control of p23 interaction (Supplementary Fig. 9b). We also performed a molecular docking modelling simulation using the X-ray crystal structure of the p23 functional domain, and identified a potential binding site on the surface of p23 that could reasonably accommodate AIL binding (Fig. 5c and Supplementary Fig. 9c). In addition, both treatment with AIL and CEL downregulated the protein level of AR rather than the chaperones HSP90 and HSP70 (Supplementary Fig. 10a), indicating that AIL and CEL might share a similar mechanism. Furthermore, p23 knockdown ablated the ability of AIL treatment to induce cell growth arrest (Supplementary Fig. 10b). Also, overexpression of p23 dose-dependently rescued AIL-mediated cell proliferation inhibition (Supplementary Fig. 10e), suggesting that p23 might be a critical target of AIL. Besides, we found that AIL indeed suppressed the activities of both glucocorticoid receptor and progesterone receptor (Supplementary Fig. 7g), suggesting that AIL is not specific in targeting AR since p23 has different client proteins. However, compared with AR, the inhibition of glucocorticoid receptor and progesterone receptor by AIL is less sensitive. For example, 0.4 μM AIL resulted in 70% inhibition of AR-induced reporter activities, but AIL just blocked the progesterone receptor and glucocorticoid receptor-induced reporter activities by about 30% (Supplementary Fig. 7g).

To investigate whether AIL suppressed the functioning of continually active AR lacking the LBD, we performed RNA-seq after treating LNCaP cells with or without AIL in the absence or presence of AR_1–651_. Indeed, as shown in Fig. 5d (top) and Supplementary Data 1, AIL strongly suppressed AR_1–651_-induced gene expression, supporting the potential therapeutic use of AIL in CPRC. Those genes not only included the classic androgen-regulated genes for example, *KLK3*, *FKBP5* and *NKX3.1* (indicated by red), but also involved other non-classic androgen-induced genes for example, *MYCBP*, *WNT10A*, *CDK2* (indicated by black), indicating that AR mutations causing LBD loss might lead to extra transcriptional functions and contribute to drug resistance. Gene Ontology analysis (Fig. 5d, bottom; Supplementary Methods) demonstrated that AR_1–651_-induced genes were involved in cell cycle, proliferation and cell adhesion, suggesting that AR lacking the LBD has oncogenic functions.

To sum up, all these data indicate that AIL prevented the interaction of p23 and HSP90 and decreased the interaction between AR and the chaperones, resulting in the ubiquitination of AR. Consequently, AR was degraded by the proteasome, AR target gene expression declined and PCa growth was blocked (Fig. 5e).

### Evaluation of AIL pharmacokinetics and CYP inhibition

**Figure 6:**
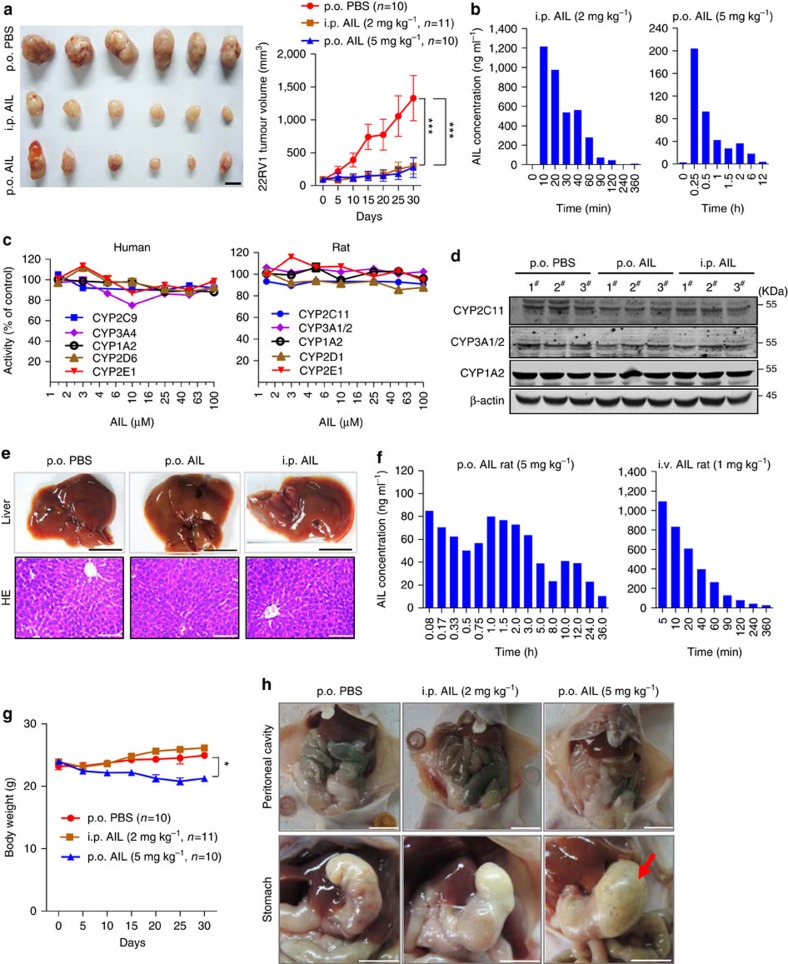
Pharmacokinetic studies of AIL. (**a**) 22RV1 tumour-bearing mice were treated with p.o. AIL or i.p. AIL and the tumour volumes were measured every 5 days; p.o. PBS served as control. Images show tumours at harvest (30 days after treatment). Scale bar, 1 cm. (**b**) The concentration of AIL in plasma was determined by LC-MS/MS following the last drug administration of p.o. AIL or i.p. AIL in tumour-bearing mice. (**c**) Effect of AIL on the activities of rat or human liver cytochrome P450 (CYP) enzymes *in vitro*. Rat liver microsomes or human liver microsomes were incubated with various concentrations of AIL and the activities of indicated CYP enzymes were measured. (**d**,**e**) The expression of CYP2C11, CYP3A1 and CYP1A2 in representative livers of the mice treated with p.o. AIL or i.p. AIL or vehicle control was measured by western blotting analysis (**d**) and the livers were photographed and stained with HE (**e**). Scale bars: the black scale bar is 1 cm (top) and the white scale bar is 50 μm (bottom). (**f**) The concentration of AIL in rat plasma was determined by LC-MS/MS after administration of p.o. AIL or i.v. AIL. (**g**) 22RV1 tumour-bearing mice were treated with p.o. AIL or i.p. AIL and mouse body weight was measured every 5 days. (**h**) Intestines and stomachs of the mice were dissected and images were taken at the end of AIL administration. Scale bar, 1 cm. Data represent the mean±s.d.; *P<0.05, **P<0.01, ***P<0.001 by one-way ANOVA followed by Bonferroni multiple comparison test.

Compounds with good absorption, distribution, metabolism, excretion and toxicity (ADME/Tox) properties are likely to increase the odds of drug discovery success. Since AIL was pharmacologically potent against CRPC in animal models, we evaluated the drug-like properties of AIL. Both oral (p.o.) and intraperitoneal (i.p.) administration of AIL were highly efficient in animal models. As shown in Fig. 6a and Supplementary Fig. 11, compared with the control group, i.p. administration (2 mg kg^−1^ per day AIL) and p.o. administration (5 mg kg^−1^ per day AIL) reduced the tumour volume of MDV3100-resistant 22RV1 xenografts by 77.5%. We noted a modest decrease in mouse body weight in the p.o. treated group (Fig. 6g), which was caused by neither overdose nor liver toxicity (Fig. 6e), but rather by AIL-induced stomach injury (Fig. 6h).

Next we determined the pharmacokinetics of AIL based on the pharmacodynamic efficiency of AIL in 22RV1 xenografts, because the pharmacokinetics–pharmacodynamic model of AIL could confirm the dose levels and drug exposures necessary for AIL to achieve potent antitumour activity *in vivo*. The concentration of AIL in nude mouse plasma was 1,216.2 ng ml^−1^ at 10 min after i.p. administration. This concentration far exceeded its effective concentration *in vitro* (IC_50_=69 nM, 25.94 ng ml^−1^), although the minimal effective concentration *in vivo* is unknown. The period that the concentrations of AIL in the plasma remained above the *in vitro* IC_50_ lasted for up to 2 h (44.83 ng ml^−1^) (Fig. 6b).

In the p.o. administration group, the plasma AIL concentration reached 203.9 ng ml^−1^ at 15 min after administration (Fig. 6b). The period that the AIL plasma concentration remained above IC_50_ *in vitro* was about 6 h because of the absorption process. Moreover, the p.o. exposure was lower than the i.p. exposure after dose normalization because of intestinal absorption as well as first-pass metabolism. Since the concentration of AIL remaining in the plasma immediately before the next administration was 1.43 ng ml^−1^ for the i.p. group and 1.84 ng ml^−1^ for the p.o. group, respectively, the efficacy of AIL *in vivo* might not last for the whole 24 h treatment interval time.

The preclinical pharmacokinetics of AIL were also evaluated in Sprague–Dawley rats (Supplementary Methods). Our previous studies have shown that the pharmacokinetic profiles of AIL in rats after intravenous (i.v.) administration exhibit linear pharmacokinetics^37^. Here we found that AIL was absorbed quickly, eliminated rapidly and distributed widely in tissues after oral administration (Fig. 6f and Table 1). Moreover, the oral bioavailability of AIL was 25.7%, which was well within the range of acceptable bioavailability (>20%), suggesting that AIL could be a potential drug candidate in clinical trials.

In addition, the effect of AIL on the activity of CYP enzymes was evaluated. As shown in Fig. 6c, AIL (1.25 to 100 μM) had no significant inhibitory effects on the main CYP isoforms (CYP1A2, 2C9/11, 2D1/6, 2E1 and 3A1/2/4) in humans and rats. Finally, we noticed that AIL did not exert obvious hepatotoxicity or significant influence on the expression of CYP2C11, CYP3A1/2 and CYP1A2 in the livers of mice (Fig. 6d,e).

## Discussion

AR mediates transcriptional programs in CRPC distinctly^38^. Current therapies have concentrated on the androgen-dependent activation of AR through its LBD, but do not provide a continuing clinical benefit for patients with CRPC and presumably fail due to multiple mechanisms including the expression of a constitutively active splice variant AR lacking the LBD. These AR-Vs can signal in the absence of ligand and are therefore resistant to LBD-targeting AR antagonists or agents that repress androgen biosynthesis^13^,^14^,^39^.

In this work, we identified a natural compound AIL that potently blocked the activities of ligand-induced full-length AR and constitutively active truncated AR which lacks the LBD. Moreover, this compound reduced the expression of both the full-length AR and the truncated AR *in vitro* and *in vivo*. Furthermore, AIL was able to inhibit MDV3100-resistant AR-Vs expressing PCa. Notably, not only i.p. administration but also p.o. administration of AIL had excellent efficiency for blocking the growth of CRPC xenografts. In pharmacokinetic studies, AIL exhibited good solubility in water and good bioavailability (>20%). In addition, AIL effectively suppressed CRPC tumour growth, despite not reaching a steady state of plasma drug concentration during the course of treatment. The stomach injury we observed may be attributable to gastrointestinal toxicity of AIL after oral administration, which is likely to be dosage-dependent. Thus, we speculate that if we shorten the treatment time interval or reduce the dosage of AIL, it would become even more effective and less toxic. In addition, we also addressed some key safety issues of AIL, such as CYP inhibition and hepatotoxicity. *In vitro* CYP inhibition data are particularly important during drug discovery for providing early warning of potential safety issues and for planning human clinical studies. Hence, the US Food and Drug Administration (FDA) recommended that CYP-associated metabolic studies *in vitro* should be performed. The current study showed that AIL had no obvious inhibitory effects on the main CYPs in humans and rats, including CYP1A2, CYP2C9 (human)/2C11 (rat), CYP2D1 (rat)/2D6 (human), CYP2E1 and CYP3A1/2 (rat)/3A4 (human) isoforms. In addition, AIL did not influence the expression of CYP enzymes and had no significant hepatotoxicity after a 5-day administration in the present study. Therefore, AIL would have a low potential to cause possible toxicity and drug–drug interactions involving CYP enzymes, suggesting a sufficient safety window for its putative use as a promising anticancer agent. Meanwhile, various physicochemical properties of AIL were calculated on the ACD/I-Lab and the results showed that the physiochemical parameters of the natural compound AIL met with ‘Lipinski's Rule of Five' (Supplementary Methods). Indeed, compounds possessing properties that exceed the Lipinski rules tend to have low oral bioavailability. Our results suggest that, if potential gastrointestinal toxicity can be overcome through dosage modulation, AIL can be developed as a potential drug candidate with various drug formulations because of its ideal solubility and bioavailability.

This study also explored the mechanism of AIL-induced AR degradation. We found that AIL disrupted the interaction between AR and the chaperones HSP90, HSP70 and HSP40, and consequently AR was ubiquitinated and degradated through the proteasome-mediated pathway. When not bound to ligand, AR resides in the cytosol bound to the foldosome, a complex of heat shock, chaperone and co-chaperone proteins including HSP90, HSP70, HSP40 and p23, among others^24^. The HSP90 dimer undergoes an ATP-driven reaction cycle. Various cofactors were regulated in this cycle: CDC37, which delivers certain kinase substrates to HSP90 and inhibits the ATPase activity; HOP, which reversibly links together the protein chaperones Hsp70 and Hsp90; p23, which stabilizes the dimerized form of HSP90 before ATP hydrolysis^36^; and HDAC6, which mediates acetylation/deacetylation of HSP90 (ref. 40). Inhibiting the chaperone HSP90 causes AR instability or blocks nuclear translocation^41^,^42^,^43^. Since AIL did not bind to HSP90 or affect chaperone expression, our results suggest that AIL is not an ATP competitive inhibitor of HSP90 like 17-AAG. However, AIL could bind to p23 protein which is very important for the stabilization of the HSP90-complex^36^ and AIL prevented the interaction of HSP90 with p23. Given that AIL was able to bind to p23 and knockdown of p23 substantially prevented AIL-induced cell growth arrest (Fig. 5b and Supplementary Fig. 10b; Supplementary Methods), we propose that AIL induces AR degradation through binding to p23 and disrupting the HSP90-client complex. Furthermore, constitutively active AR variant expression does not confer resistance to AIL. Indeed, recent papers have shown that constitutively active AR variants played their roles independently of the HSP90 chaperone but did not confer resistance to HSP90 inhibitors^44^,^45^, indicating that the mechanisms of AIL also include HSP90 complex inhibition. Not surprisingly, AIL also suppressed the activities of other nuclear receptors including progesterone and glucocorticoid receptor (Supplementary Fig. 7g), indicating that repression of glucocorticoid and progesterone receptor signalling might contribute the therapeutic efficiencies of AIL in CRPC. At higher concentrations (up to 10 μM as well as 50 μM), AIL also significantly decreased the cell growth of PC3 and DU145 (Fig. 1e and Supplementary Fig. 2), which might be caused by the degradation of other p23 clients (AKT and Cdk4). Indeed, prostate cancer cells that express AR showed greater sensitivity to inhibition of growth by AIL at lower concentration, suggesting the degradation of AR by AIL plays a major role in inhibiting cell growth of AR-positive prostate cancer cells at low concentrations of AIL. Alternatively, the degradation of AR and other clients including AKT and Cdk4 may have induced synthetic lethality by blocking multiple signal pathways in AR positive cells, rendering AR positive cell lines sensitive to AIL. Knockdown of AR achieved only about 30% growth inhibition, whereas p23 knockdown was more effective in inhibiting 22Rv1 cell growth (Supplementary Fig. 10b), suggesting that other downstream targets of AIL mediated by the inhibition of p23, such as AKT, Cdk4 or others are important for prostate cancer cell growth inhibition. Therefore, we conclude that targeting p23 is the major mechanism of AIL. Meanwhile, AIL-induced AR degradation is at least a critical mechanism of AIL-dependent cell growth inhibition in prostate cancer. Since overexpression of p23 could not totally rescue the AIL-induced cell growth inhibition (Supplementary Fig. 10e), we conclude that p23 also has other potential targets including protein synthesis^46^.

In fact, how AIL regulates the molecular conformation of p23 and prevents the interaction of p23 with HSP90 remains undetermined in our work. Clarifying the mechanism of AIL remains to be further investigated.

p23 is able to increase the AR protein level and AR transcriptional activity which is independent of its role in the HSP90 foldosome complex^30^. Significantly, p23 expression is implicated in resistance to HSP90 inhibitors^28^, and plays a role in PCa metastasis. Consequently, inhibition of p23 is likely to counteract CRPCs that have developed resistance to HSP90 inhibitors, and AIL may serve to synergistically enhance the efficacy of HSP90 inhibition in ablating CRPC in addition to its efficacy as a solitary agent against CRPC. CEL that effectively inhibits prostate cancer cells^47^,^48^ has been reported to inhibit p23 function and to bind to three cystine residues of p23: Cys-40, Cys-58 and Cys-75 (ref. 49). Importantly, our molecular modelling indicates that AIL binds to a different region of p23 (Fig. 5c; Supplementary Methods), suggesting that AIL has the potential to synergize with CEL in inhibiting p23. Finally, p23 has also been implicated in breast cancer lymph node metastasis and drug resistance^31^, highlighting the potential value of AIL in treating multiple cancer types.

In conclusion, we screened and characterized AIL, a novel compound with excellent drug-like characteristics that is able to overcome MDV3100-resistance in prostate cancer cell lines. AIL was efficacious in suppressing the growth and metastasis of CRPC via targeting p23. As a result, AIL can be considered a new potential drug candidate for prostate cancer, and it is worthy of further research and investigation.

## Methods

### Cell culture

Prostate cancer cell lines c4-2b, LAPC4 and normal prostate epithelial cell line RWPE-1 used in this study were kindly provided by Dr Ying-Hao Sun (Department of Urology, Changhai Hospital, Shanghai, China). Other human prostate cancer cell lines were purchased from the Cell bank of the Chinese Academy of Science. The cell lines were authenticated by short tandem repeat analysis and mycoplasma contamination was tested by the PCR Mycoplasma Detection Set (Takara, Otsu, Japan). 293T cells were routinely maintained in DMEM (Gibco), while prostate cancer cells were cultured in RPMI 1640 (Gibco). Media were supplemented with 10% FBS (BioWest, catalogue no. S1580-500) and 1% penicillin/streptomycin unless otherwise specified. RWPE-1 was cultured in serum-free medium (Invitrogen, Carlsbad, CA).

### Dual luciferase screening assay

For dual luciferase screening assay, prostate cancer cells were transfected with MMTV-luc, Renilla-luc (phRL-TK, Promega), AR or AR_1-651_ (vector: pFLAG-CMV-1) plasmids (provided by Dr Jie-Min Wong)^50^ using lipofectamine 2000 (Invitrogen) according to the manufacturer's instructions. After transfection for 24 h, the transfected cells were treated with DHT (Sigma, catalogue no. A8380) or DHT with compounds for 12 h. Renilla and firefly activities were then determined by luminometry using the Dual-Luciferase Reporter Assay System (Promega) and the ratio calculated. Results were expressed as the ratio of firefly to Renilla luciferase activity.

### Quantitative real-time PCR

Cells were cultured with RPMI 1640 with 5% charcoal dextran-treated FBS for 5 days before treatment with R1881 (Sigma, catalogue no. R0908) alone or R1881 and AIL for 12 h. Total RNA was extracted using TRIzol (Takara, Japan) according to the manufacturer's instructions. One microgram of total RNA was used for complementary DNA synthesis using a cDNA reverse transcription kit (Takara, Japan). Real-time PCR was performed in triplicate using gene-specific primers on a Stratagene Mx3005P PCR system (Agilent Technologies) machine. The mRNA expression levels were normalized to β-actin expression or GAPDH. All analysis was performed using Microsoft Excel 2010 and GraphPad Prism 5 software. The gene-specific primers are listed in Supplementary Table 2.

### SRB assay

For SRB assay, cells were cultured in complete RPMI 1640 and incubated with indicated concentrations of AIL or cells were maintained in fresh phenol red-free RPMI 1640 medium with 5% charcoal-stripped FBS (c-FBS; Wisent), 1 nM DHT and indicated compounds. After 48 or 72 h, the cells were then fixed and the cell growth was detected with the SRB assay^51^. AIL was purchased from Shanghai Zhanshu Chemical Technology, Co., Ltd (Shanghai, China). BIC and MDV3100 (MDV) were purchased from Selleckchem.

### Cell colony formation assay

For colony formation assay, prostate cancer cells were incubated with indicated concentrations of AIL in complete RPMI 1640 for 2 weeks and then cells were fixed with 4% paraformaldehyde and stained with crystal violet. Colonies were visualized under a microscope, and all of the fields were imaged and counted. Colony formation as a percentage of vehicle control for each cell line is presented.

### Western blotting

Cells were treated as described in the corresponding section of Results and then lysed by boiling for 10 min in sample buffer (2% SDS, 10% glycerol, 10% β-mercaptoethanol, bromphenol blue and Tris-HCl, pH 6.8). Lysates were fractionated on polyacrylamide gels and transferred to nitrocellulose. The blots were probed with specific antibodies followed by secondary antibody then membranes were examined using the LI-COR Odyssey infrared imaging system (LI-COR Biotechnology, Lincoln NE). The AR (N20, sc-816 and H280, sc-13062; 1:1,000), HSP90 (sc-7947; 1:1,000), and Cdk4 (sc-260; 1:1,000) antibodies were purchased from Santa Cruz Biotechnology (Santa Cruz, CA). The HSP70 (1776-1; 1:5,000), HSP40 (3532-1; 1:1,000) and ubiquitin (1646-1; 1:1,000) antibodies were purchased from Epitomics (Burlingame, CA). p23 (ab92503; 1:1,000) and Hop (ab126724; 1:1,000) antibodies were purchased from Abcam (Cambridge, MA). Akt (4691; 1:1,000) and HDAC6 (7558; 1:1,000) antibodies were purchased from Cell Signaling Technology (Danvers, MA). CDC37 (4222S; 1:1,000) antibody was purchased Biogot Biotechnology Co., Ltd (Shanghai, China). The β-actin antibody (1:10,000) was purchased from Sigma (St Louis, MO). The secondary antibody was conjugated with IRDye 680/800 (Millennium Science; 926–32221, 926–32210; 1:10,000). Uncropped western blots are shown in Supplementary Fig. 13.

### Co-immunoprecipitation

22RV1 and LNCaP cells were treated with or without AIL in the presence of 10 μM MG132. After 24 h, cells were washed with cold PBS and collected in immunoprecipitation buffer (0.1% Triton X-100, 2 mg ml^−1^ aprotinin, 100 mg ml^−1^ PMSF, 100 mM NaCl in 50 mM Tris-HCl, pH 7.2). The lysate was lysed for 1 h at 4 °C and centrifuged at 16,000*g*. The supernatants were incubated with 2 μg antibody to AR (Santa Cruz, H280), HSP90 (Santa Cruz, sc-7947) or HSP70 (Epitomics, 1776-1) with 20 μl of protein A/G (Abmart) and rocked for 2.5 h at 4 °C. The protein A/G beads were pelleted and washed three times with immunoprecipitation wash buffer. The precipitates were resolved on SDS– polyacrylamide gel electrophoresis gel and subjected to western blot analysis.

### *In vivo* subcutaneous tumour growth xenograft models

BALB/c-nude mice (6–8-weekold, male) were purchased from the Sino-British Sippr/BK Lab Animal Co., Ltd (Shanghai, China) and maintained under pathogen-free conditions. The animal use protocol was approved by the Institutional Animal Care and Use Committee of East China Normal University. The 22RV1, LNCaP and VCaP xenograft tumour models were developed by injecting 3 × 10^6^ 22RV1 cells or 5 × 10^6^ LNCaP or VCaP cells in suspension into the right flank of a BALB/c-nude mouse; cells were suspended in 100 μl PBS or 50% matrigel (LNCaP and VCaP), respectively. Specifically for LNCaP and VCaP cells, continuous release testosterone pellets (15 mg testosterone per pellet, Sigma-Aldrich) were implanted subcutaneously to stimulate the growth of LNCaP and VCaP xenografts. Tumour nodules were allowed to grow to a volume of about 100 mm^3^ before initiating treatment. Tumour-bearing BALB/c-nude mice were randomly assigned to three groups and treated with the indicated compound or drug. The tumour volume and mouse body weight were measured twice a week. The tumour volume was calculated using the following equation: tumour volume (*V*)=length × width × width × 0.52.

### Orthotopic castration-resistant prostate cancer model

For orthotopic castration-resistant prostate cancer xenografts, male BALB/c-nude mice (8–9 weeks of age) were anaesthetized using 150 mg kg^−1^ 2, 2, 2-tribromethanol plus 350 mg kg^−1^ tert-amyl alcohol and then 5 × 10^5^ 22RV1-luc cells suspended in 30 μl 50% matrigel were surgically injected into the dorsolateral prostate lobes. One week after injection, the tumour-bearing mice were castrated and randomly assigned to three groups. A week later, animals were intraperitoneally injected with AIL (2 mg kg^−1^), MDV (10 mg kg^−1^) or DMSO (as controls). Prostate tumour growth and local metastasis were monitored weekly using the IVIS Imaging System (Xenogen Corporation, Alameda, CA). Images and measurements of bioluminescent signals were acquired and analysed using Living Image and Xenogen software^52^.

### Histology and immunohistochemistry

Tumours or mouse tissue samples were immediately fixed in 10% neutral buffered formaldehyde for 24 h, progressively dehydrated in solutions containing an increasing percentage of ethanol (75, 85, 95 and 100%, v/v), and embedded into paraffin blocks. For immunohistochemical (IHC) staining, sections were cut from the paraffin blocks and IHC was carried out using anti-Ki-67 (1:250), and anti-AR (1:50; N-20) as primary antibodies. Samples were stained with haematoxylin–eosin (HE) to indicate nucleus and cytoplasm, respectively.

### Geldanamycin-FITC fluorescence polarization assay

Fluorescence polarization assay^53^ measurement of binding affinities between AIL and p23 as well as HSP90 was used to confirm whether AIL inhibited fluorescein-conjugated geldanamycin (FITC-GA) binding to the ATPase site of the HSP90α isoform. Detailedly, FITC-GA (Invivogen, catalogue no. ant-fgl-1) was dispensed into wells containing AIL at a final concentration of 0.16 nM FITC-GA. HSP90α recombinant protein (BPS, catalogue no. 50290) in buffer (50 mM KCl, 5 mM MgCl_2_, 20 mM HEPES, pH 7.3−7.5, 0.1% CHAPS (Sigma, catalogue no. C5070), 0.1% bovine gamma-globulin (Sigma, catalogue no. G7516) and 2 mM dithiothreitol (Sigma, catalogue no. 646563) was then added to the well at final concentration of 30 nM. For IC_50_ determination, 100 μM AIL was serially diluted 1:4 by transferring 20 μl into 60 μl of 100% DMSO into successive wells for a total of 10 final concentrations. As a positive control, 10 μM 17-AAG (Selleckchem, catalogue no. S1141) was serially diluted 1:10 by transferring 10 to 90 μl of 100% DMSO in the next well repeatedly for a total of 10 final concentrations. The assay plate was covered and incubated at 4 °C overnight. Data were collected on Victor-3 with the setting Ex480/Em535. mP values were converted to percent inhibition values. Percent inhibition=(sample RLU−min)/(max−min) × 100%. ‘min' means the mP of no enzyme control and ‘max' means the mP of DMSO control. Data were graphed in MS Excel and the curves were fitted by XLFit Excel add-in version 4.3.1.

### AIL-p23 docking studies

The protein structure of p23 was obtained from Protein Data Bank (PDB ID: 1EJF) and the PDB file was processed by removing water molecules and cations for the next docking step. Docking studies were performed by using Autodock Vina 1.1.2, and all images were generated in UCSF Chimera 1.8. The protein structure of p23 was obtained from Protein Data Bank (PDB ID: 1EJF) and the PDB file was processed by removing water molecules and SO_4_^2−^ for the next docking step. Docking studies were performed by using Autodock Vina 1.1.2, and all images were generated in UCSF Chimera 1.8. The active site was similar to the reported site^49^,^54^,^55^. The correlative parameters were listed in Table 2 and other parameters chosen were: num_modes=9 and exhaustiveness=16. The lowest energy conformation was chosen for binding model analysis.

### Pharmacokinetic studies and CYP-associated metabolic studies

Pharmacokinetic studies *in vivo*^37^ and CYP-associated metabolic studies *in vitro*^56^ were performed using the method reported previously in our laboratory. In this study, the effects of AIL on CYP activities were investigated using rat and human liver microsomes, employing phenacetin (CYP1A2), tolbutamide (CYP2C9/11), dextromethorphan (CYP2D1/6), chlorzoxazone (CYP2E1) and testosterone (CYP3A2/4) as the probe substrates. They were analysed on an Agilent 1260 series instrument with DAD detection and separated by an Agilent ZORBAX Eclipse XDB-C18 column (4.6 × 150 mm, 5 μm) with a guard column in the respective gradient elution procedure. The incubation system, sample preparation and chromatography conditions are as described previously^56^.

### AIL treatment of tumour-bearing BALB/c-nude mice

22RV1 xenografts were performed as descripted in ‘*In vivo* subcutaneous tumour growth xenograft models' above. After the volume of a tumour nodule reached about 100 mm^3^, tumour-bearing BALB/c-nude mice were randomly assigned to three groups and treated with 2 mg kg^−1^ AIL (intraperitoneal injection, i.p.) or 5 mg kg^−1^ (oral administration, p.o.) and the control group was orally treated with an equal volume of PBS (p.o.). Since we found that AIL was water soluble, AIL was dissolved in PBS in this experiment. After 30 days of treatment, all nude mice were subjected to retroorbital bleeding to obtain blood samples, and then were killed. Plasma samples were collected at the indicated time points after the last administration.

### HPLC-MS/MS determination of AIL concentrations

A simple and sensitive method for the determination of AIL in plasma was developed, using high-performance liquid chromatography-tandem mass spectrometry (HPLC-MS/MS). Brusatol was used as an internal standard. Separation was achieved on an Agilent Zorbax Eclipse Plus C18 column (2.1 × 50 mm, 1.8 μm; USA) with gradient elution using water–methanol as mobile phase at a flow rate of 0.2 ml min^−1^, and total run time was 7.0 min. A triple quadrupole mass spectrometer operating in the negative electrospray ionization mode with multiple reaction monitoring was used to detect AIL and IS transitions of 375.2→301.1 and 519.1→437.4, respectively. The details of this HPLC-MS/MS method and sample preparation are described in our previous study^37^.

### Statistical analysis

The statistical analysis was performed by SPSS 22.0 software. The differences between control group and experimental groups were determined by one-way analysis of variance. Since treatment and time course was investigated, two-way analysis of variance followed by *post hoc* test was also applied. Data were expressed as mean and s.d., and *P*<0.05 was considered significant. Pharmacokinetic parameters were calculated by WinNonlin software version 5.2.1 (Pharsight Corporation, Mountain View, USA) based on noncompartmental analysis.

### Data availability

RNA-seq data have been deposited into NCBI Gene Expression Omnibus with accession number GSE85541. (http://www.ncbi.nlm.nih.gov/geo/query/acc.cgi?token=kbavwmoqdrahfel&acc=GSE85541).

## Additional information

**How to cite this article:** He, Y. *et al*. Ailanthone targets p23 to overcome MDV3100 resistance in castration-resistant prostate cancer. *Nat. Commun.* **7,** 13122 doi: 10.1038/ncomms13122 (2016).

## Supplementary Material

Supplementary Information Supplementary Figures 1-13, Supplementary Tables 1-5, Supplementary Methods and Supplementary References.

Supplementary Data 1 Expression level (log2 based) of AR1-651 induced genes which inhibited by AIL.

## Figures and Tables

**Table 1:** The pharmacokinetic parameters of AIL after oral administration or intravenous injection in rats (mean±s.d.).

**Pharmacokinetic parameters**	**p.o. (5 mg kg**^**−1**^**)** ***n*****=6**	**i.v. (1 mg kg**^**−1**^**)** ***n*****=6**
*T*_1/2_ (min)	730.2±155.9	113.3±39.6
*T*_max_ (min)	23.3±31.8	—
*C*_max_ (ng ml^−1^)	87.0±16.4	—
*C*_max_ (nM)	231.1±43.6	
*C*_0_ (ng ml^−1^)	—	1,653.2±98.6
*C*_0_ (nM)		4,392.1±261.9
AUC_0–*t*_ (min ng ml^−1^)	67,324.5±7,405.3	57,874.3±6,871.1
AUC_0–∞_ (min ng ml^−1^)	79,053.9±14,616.6	61,517.4±5,986.2
Bioavailability	25.7%	—

**Table 2:** Parameters of docking studies.

	***X***	***Y***	***Z***
Centre	3.261	19.118	33.447
Size of box	42	40	44
