# Supplementary material for: Ailanthone targets p23 to overcome MDV3100 resistance in castration-resistant prostate cancer
Source: Nat Commun. 2016 Dec 13;7:13122. doi: 10.1038/ncomms13122 (PMC5159881; doi:10.1038/ncomms13122)
Supplement: Supplementary Information — Supplementary Figures 1-13, Supplementary Tables 1-5, Supplementary Methods and Supplementary References. [file ncomms13122-s1.pdf]

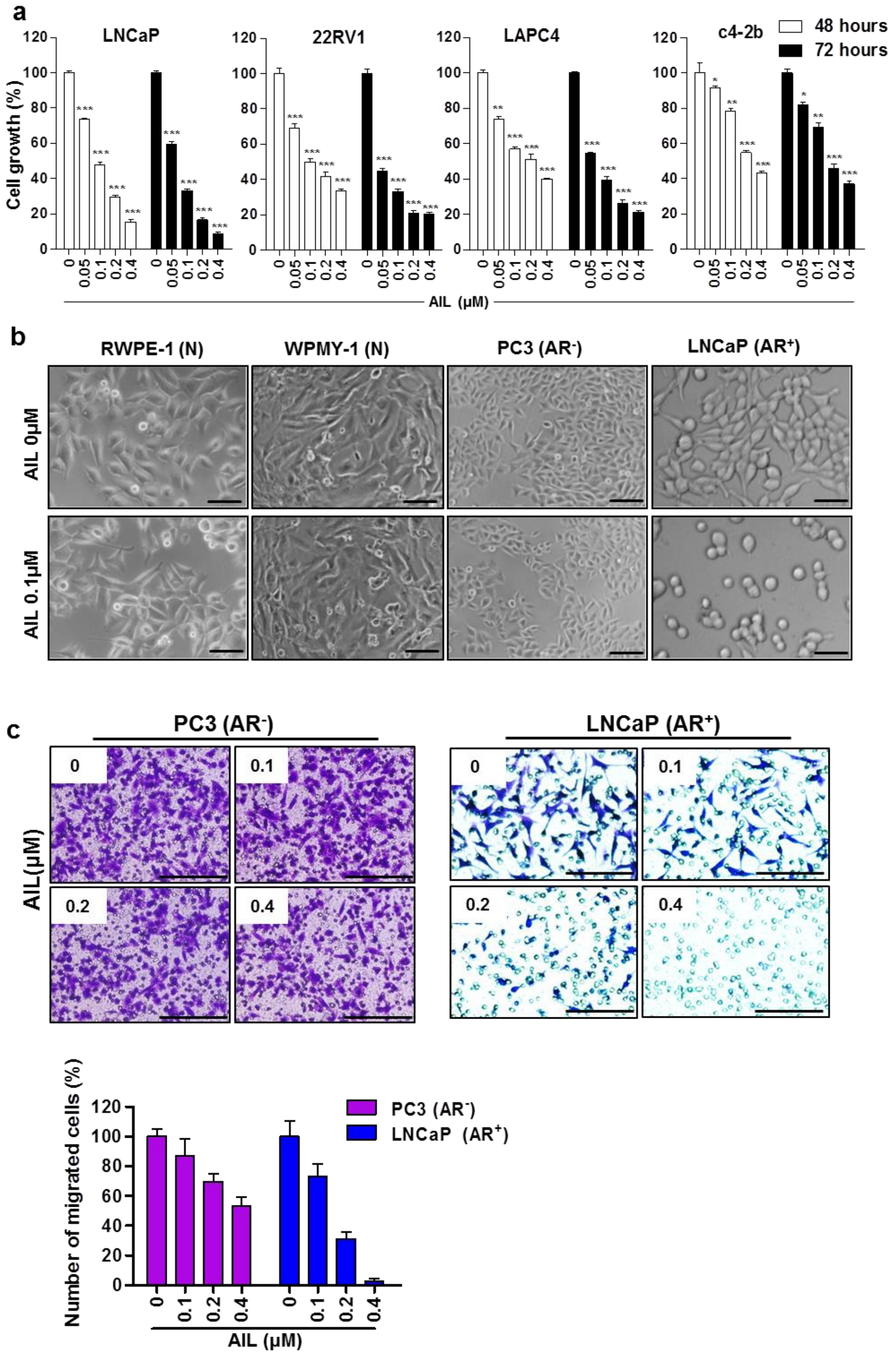

Supplementary Figure 1. AR positive prostate cancer cells are more sensitive to AIL. (**a**) LNCaP, 22RV1, LAPC4, c4-2b cells were treated with indicated concentrations of AIL for 48 h or 72 h and cell proliferation was detected with the SRB assay (n = 5). (**b**) Prostate normal cell lines RWPE-1 and WPMY-1, AR negative cell line PC3 and positive cell line LNCaP were treated with 0 or 0.1 µM AIL for 48 hours. The cells were imaged and a cropped region from each image is shown to

enable a closer view. Scale bar, 50 µm. (**c**) AR negative cell line PC3 and positive cell line LNCaP prostate cancer cells were treated with different concentrations of AIL in Transwell chambers for 18 hours and the migrated cells were imaged and counted. Scale bar, 100 µm. Data was expressed as mean ± s.d.; Student's t-tests were performed; * $P < 0.05$, ** $P < 0.01$, *** $P < 0.001$.

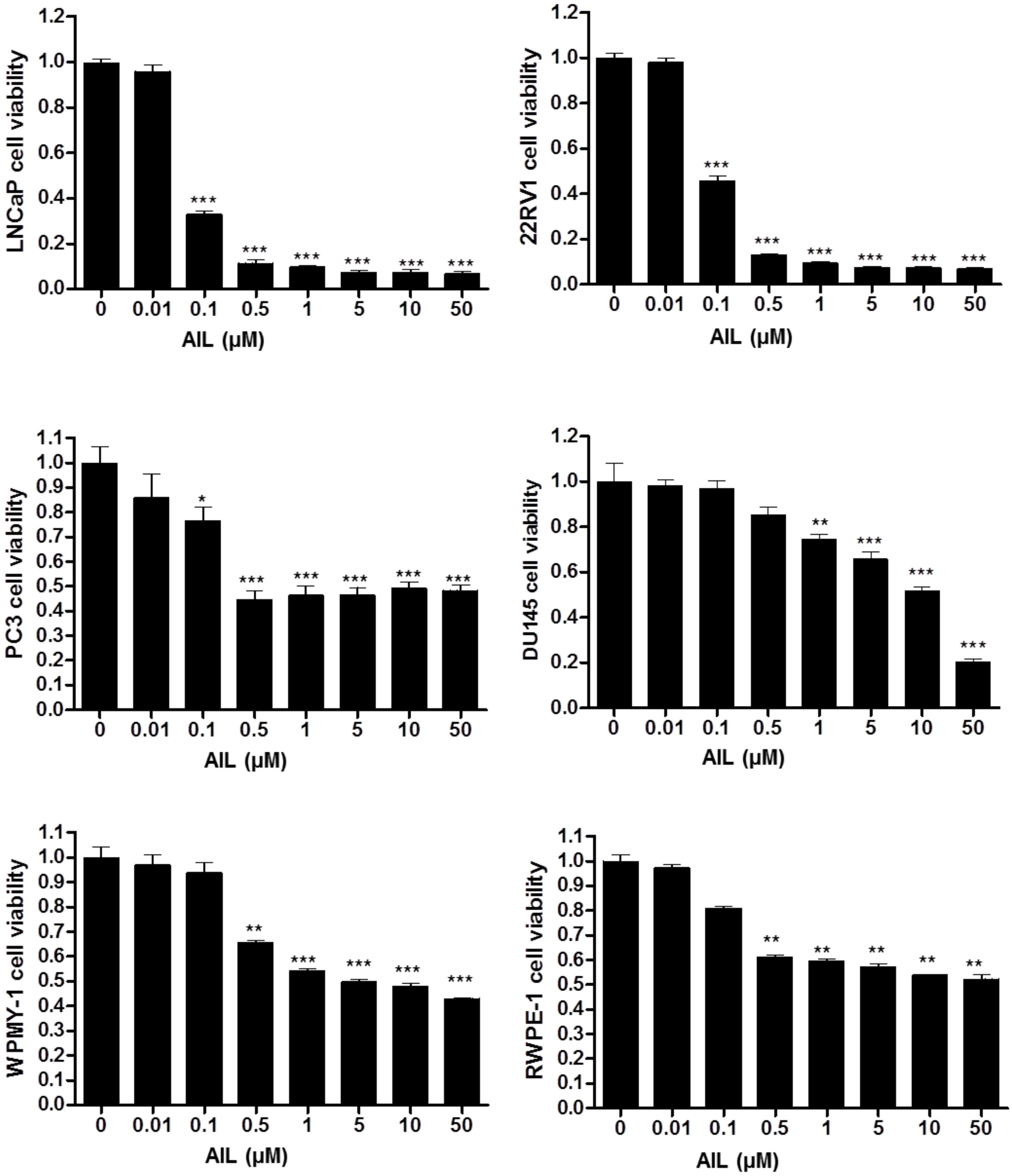

Supplementary Figure 2. Inhibitory effects of AIL on proliferation of prostate cancer cells and normal prostate cell lines. Prostate cancer cells (LNCaP, 22RV1, PC3, DU145), human prostate stromal cells WPMY-1 and the human normal prostate epithelial cell line RWPE-1 cells were treated with indicated concentrations of AIL for 48 h and the cell proliferation was detected with the SRB assay (n = 5). Data was expressed as mean ± s.d.; Student's t-tests were performed; * $P < 0.05$, ** $P < 0.01$, *** $P < 0.001$.

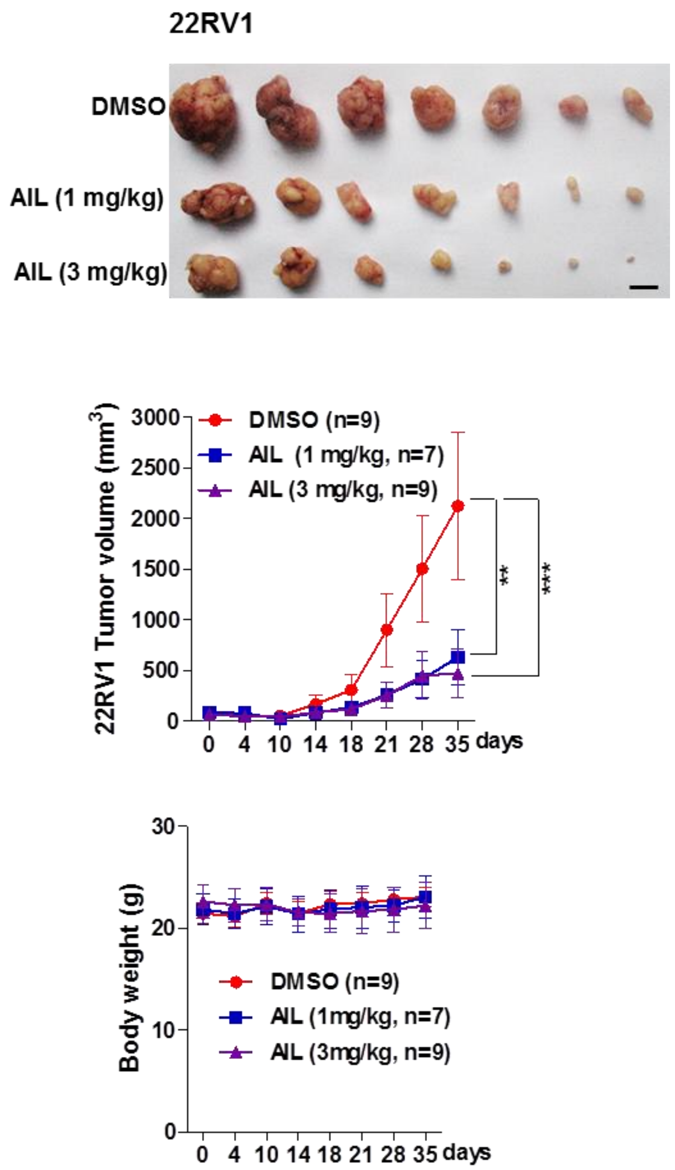

Supplementary Figure 3. Cytoreduction of 22RV1 xenografts *in vivo* by AIL. 3×10^6 22RV1 cells with 0.1 ml PBS were injected into the right flank of BALB/c-nude mice. After the volume of tumor nodules was about 100 mm^3, the mice were randomly assigned to the indicated groups and *i.p.* injected daily with DMSO (control), 1mg/kg AIL and 3mg/kg AIL, respectively. Tumor volumes and the mouse body weights were measured twice a week. The mice were sacrificed after 35 days and the tumors from each group of mice were excised and images were taken. Scale bar, 1 cm. Data represent the mean ± s.d. ** $P < 0.01$, *** $P < 0.001$ by one-way ANOVA followed by Bonferroni multiple comparison test.

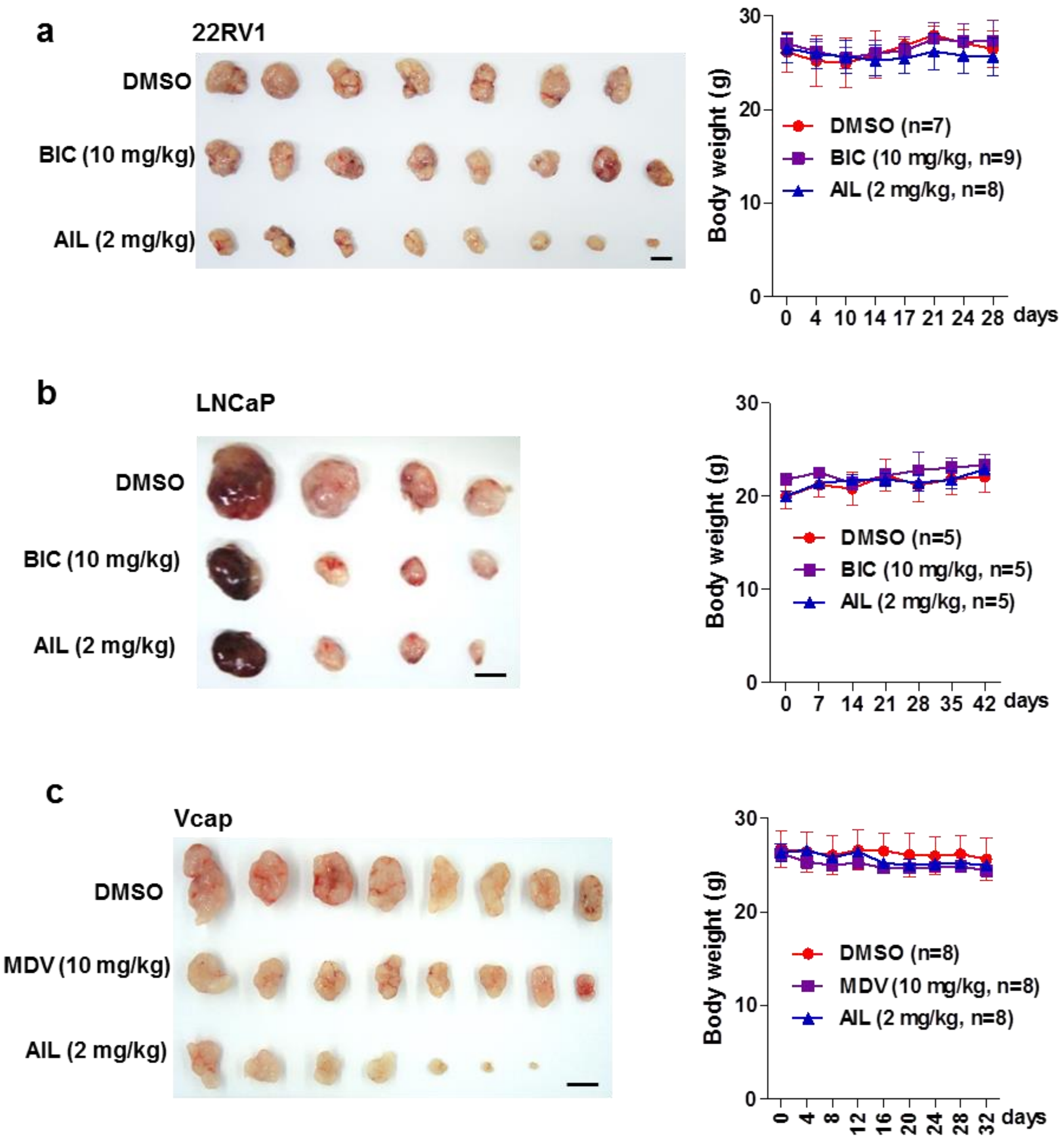

Supplementary Figure 4. Comparing the anti-tumor efficiency of AIL with AR antagonists in different xenograft models. (**a-c**) 22RV1 (a), LNCaP (b) and Vcap (c) cells were injected with 0.1 ml PBS (22RV1) or Matrigel (LNCaP, Vcap) into the right flank of BALB/c-nude. After the volume of tumor nodules reached about 100 mm^3, the mice were randomly assigned to the indicated groups and i.p. injected daily with DMSO (control), AIL, BIC, or MDV as indicated. Tumor volume and the mouse body weight were measured once a week. The mice were sacrificed after 28 days (22RV1), 49 days (LNCaP) or 32 days (Vcap) and the tumors from each group of mice were harvested and images were taken. Scale bar, 1 cm. Data represent the mean ± s.d.

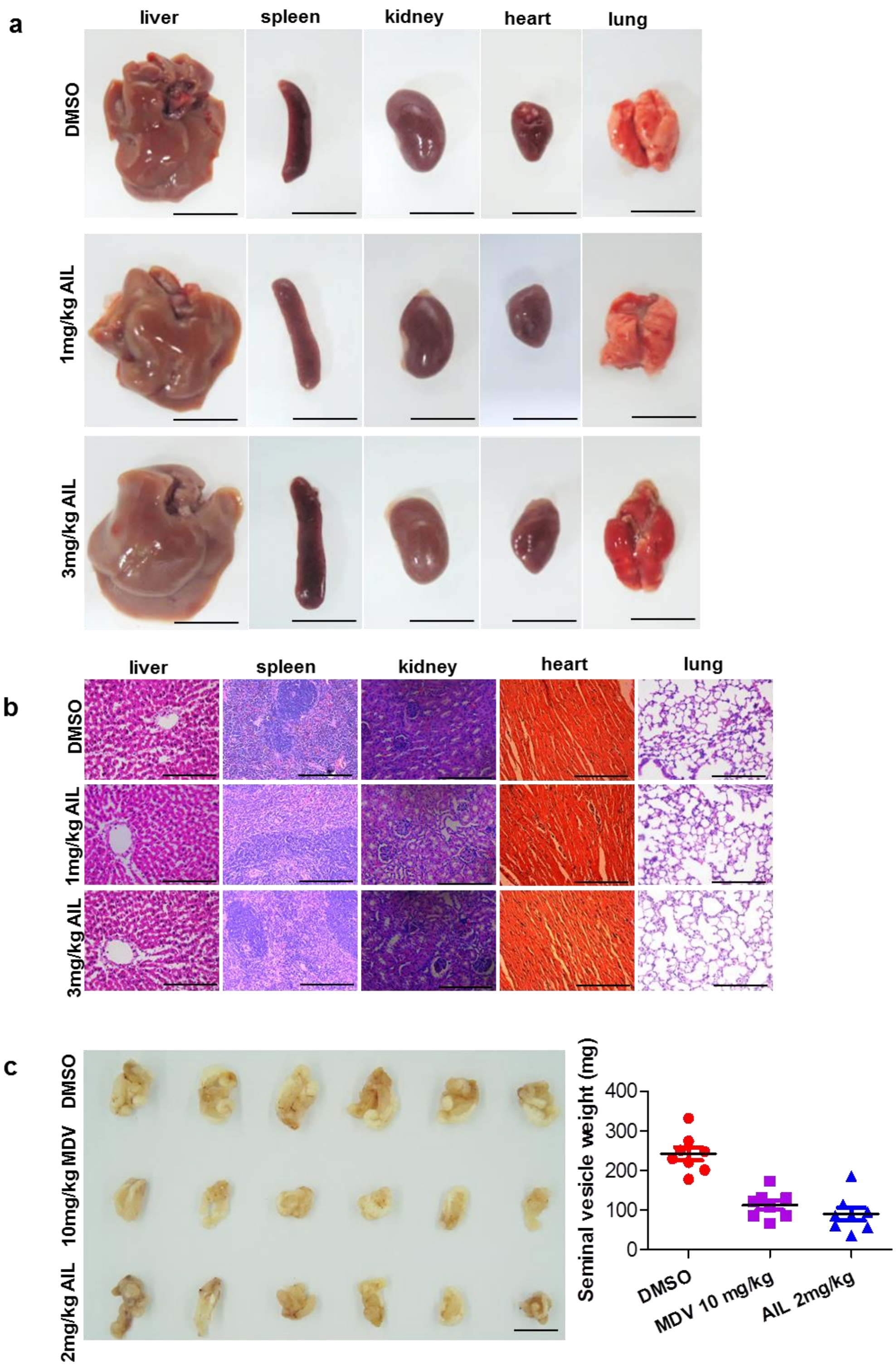

Supplementary Figure 5. *I.p.* delivery of AIL did not cause toxicity. (**a,b**) 3×10^6 22RV1 cells were injected with 0.1 ml PBS into the right flank of a nude mouse. After tumor nodules grew to a volume about 100 mm^3, the mice were randomly assigned to three groups and respectively *i.p* injected with DMSO, 1 or 3 mg/kg/day AIL. After 35 days, mice were sacrificed and organs were removed. Photographs (a) and HE-staining (b) of the representative organs from mice administered daily with

DMSO, 1 mg/kg AIL or 3 mg/kg AIL were illustrated. Tissues shown in Figure S3 were from the same animals. Scale bar, 1 cm in panel **a** and 100 µm in panel **b**. (**c**) 3×10^6 Vcap cells were injected with 0.1 ml matrigel into the right flank of a nude mouse. After tumor nodules grew to a volume about 100 mm^3, the mice were randomly assigned to three groups and respectively *i.p* injected with DMSO, 10mg/kg MDV or 2mg/kg AIL. After 35 days, mice were sacrificed and prostate and seminal vesicle tissues were excised. Photographs of the representative organs from mice administered with DMSO, 10 mg/kg MDV or 2 mg/kg AIL were illustrated. The weight of seminal vesicles was measured (Right panel). Tissues shown in Figure 2c were from the same animals. Scale bar, 1cm.

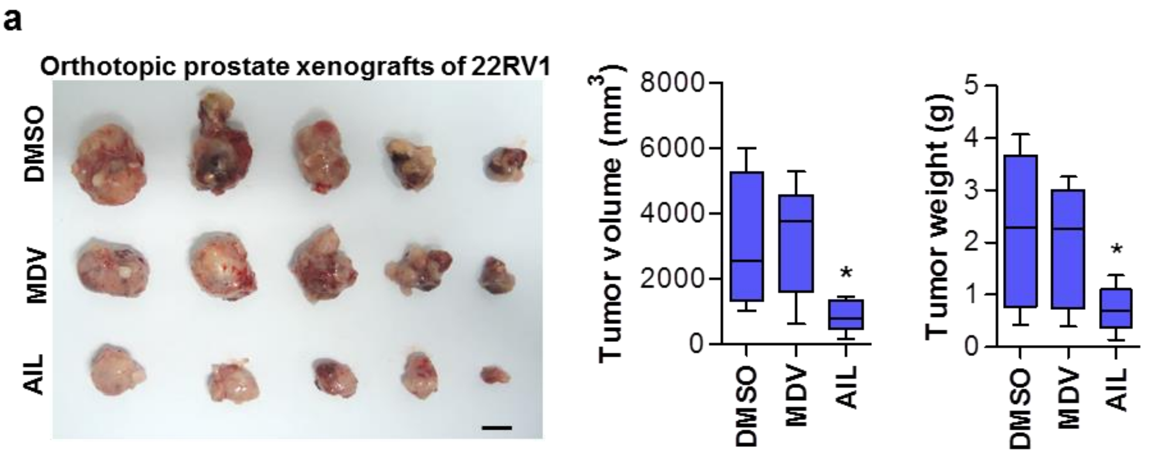

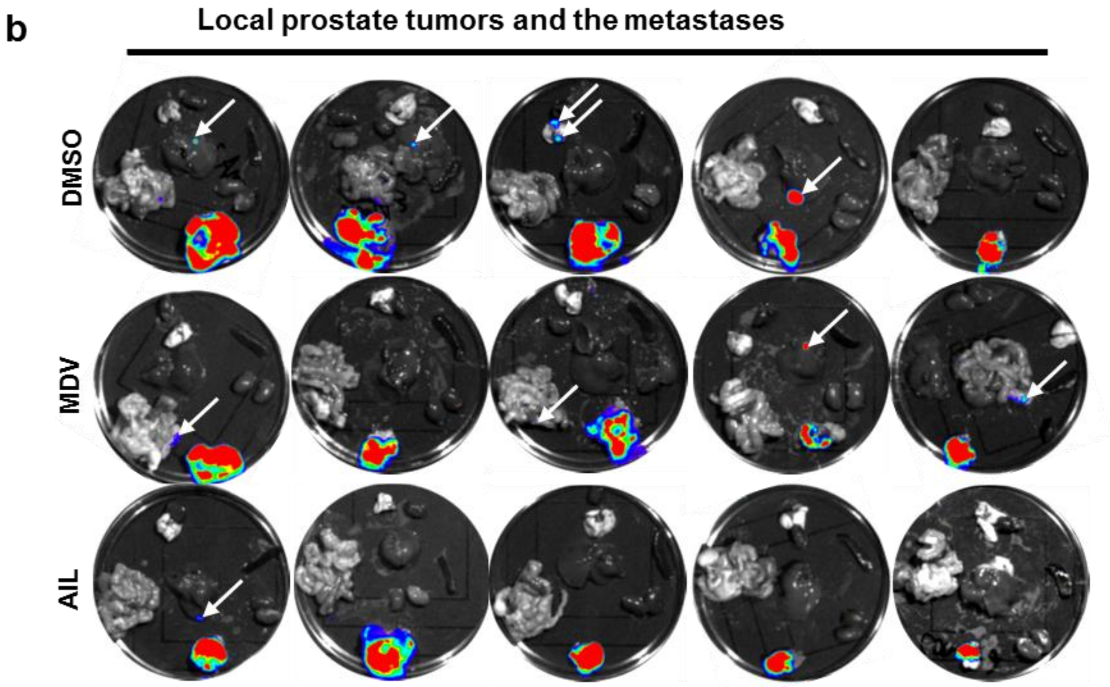

Supplementary Figure 6. AIL inhibited tumor growth and metastasis in a CRPC animal model. (**a,b**) The dorsolateral prostate of male BALB/c-nude mice was injected with 1 × 10^6 22RV1-luc cells in 30 μl matrigel. After a week, mice were castrated and injected *i.p.* with DMSO, 10 mg/kg MDV or 2 mg/kg AIL once a day. After 28 days, mice were sacrificed and the local tumors were removed and volumes and weight of the tumors were measured (a). The local tumors and viscera of the mice were imaged for counting metastatic tumors (b). Scale bar, 1 cm. Data was expressed as mean ± s.d.; Student's t-tests were performed; * $P < 0.05$.

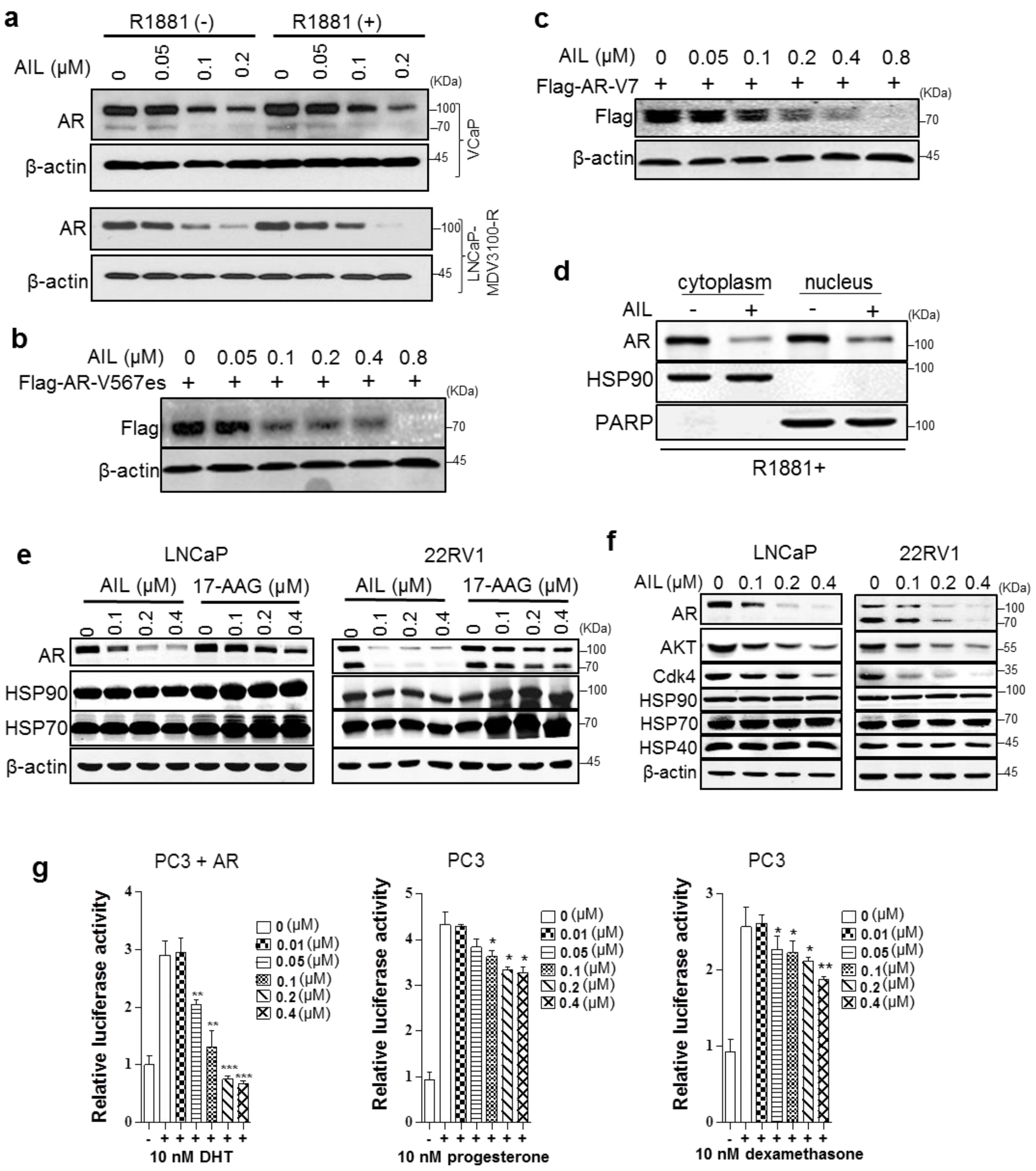

Supplementary Figure 7. Effect of AIL on the protein levels of AR and molecular chaperones. (**a**) VCaP and LNCaP-MDV3100-R cells were treated for 12 hours with indicated concentrations of AIL with or without R1881 and the AR protein level was measured by Western blotting analysis. (**b,c**) AIL decreased the protein level of AR variants. Flag-AR-V567es (b) or Flag-AR-V7 (c) plasmids were transfected into PC3 cells. After 24 hours, cells were treated with the indicated concentrations of AIL for 12 hours and cells were lysed and AR-V7 and AR-V567es protein levels were measured by Western blot analysis using Flag antibody. (**d**) AIL decreased the AR protein level in both cytoplasm and nucleus. PC3 cells were transfected with AR for 24 hours and treated with or without 0.4 μM AIL in the presence of R1881 for the next 12 hours. Nucleocytoplasmic separation was done and the protein level of AR in

cytoplasm and nucleus was measured by Western blot analysis. HSP90 is a cytoplasm marker and PARP is a nuclear marker. (**e**) HSP90 and HSP70 proteins were induced by HSP90 inhibitor 17-AAG rather than AIL. LNCaP and 22RV1 cells were treated for 12 hours with the indicated concentrations of AIL or 17-AAG. Cells were lysed and AR, HSP90 and HSP70 protein levels were measured by Western blot analysis. (**f**) AIL down-regulates the protein level of HSP90 clients but not the molecular chaperones. LNCaP and 22RV1 cells were treated for 24 hours with the indicated concentrations of AIL, cells were lysed and indicated protein levels were measured by Western blotting analysis. (**g**) Effect of AIL on the activities of androgen receptor (AR), glucocorticoid receptor (GR) or progesterone receptor (PR). PC3 cells transfected with or without AR as indicated were transiently transfected with MMTV-luc reporter plasmid and Renilla-luc plasmid, stimulated by 10 nM AR agonist DHT (left), or 10 nM PR agonist progesterone (middle), 10 nM GR agonist dexamethasone (right). Then cells were treated with different concentrations of AIL for 12 hours and the luciferase activities were measured and results were expressed as the ratio of luciferase activity. Data was expressed as mean ± s.d. of three independent assays; Student's t-tests were performed; * $P < 0.05$, ** $P < 0.01$, *** $P < 0.001$.

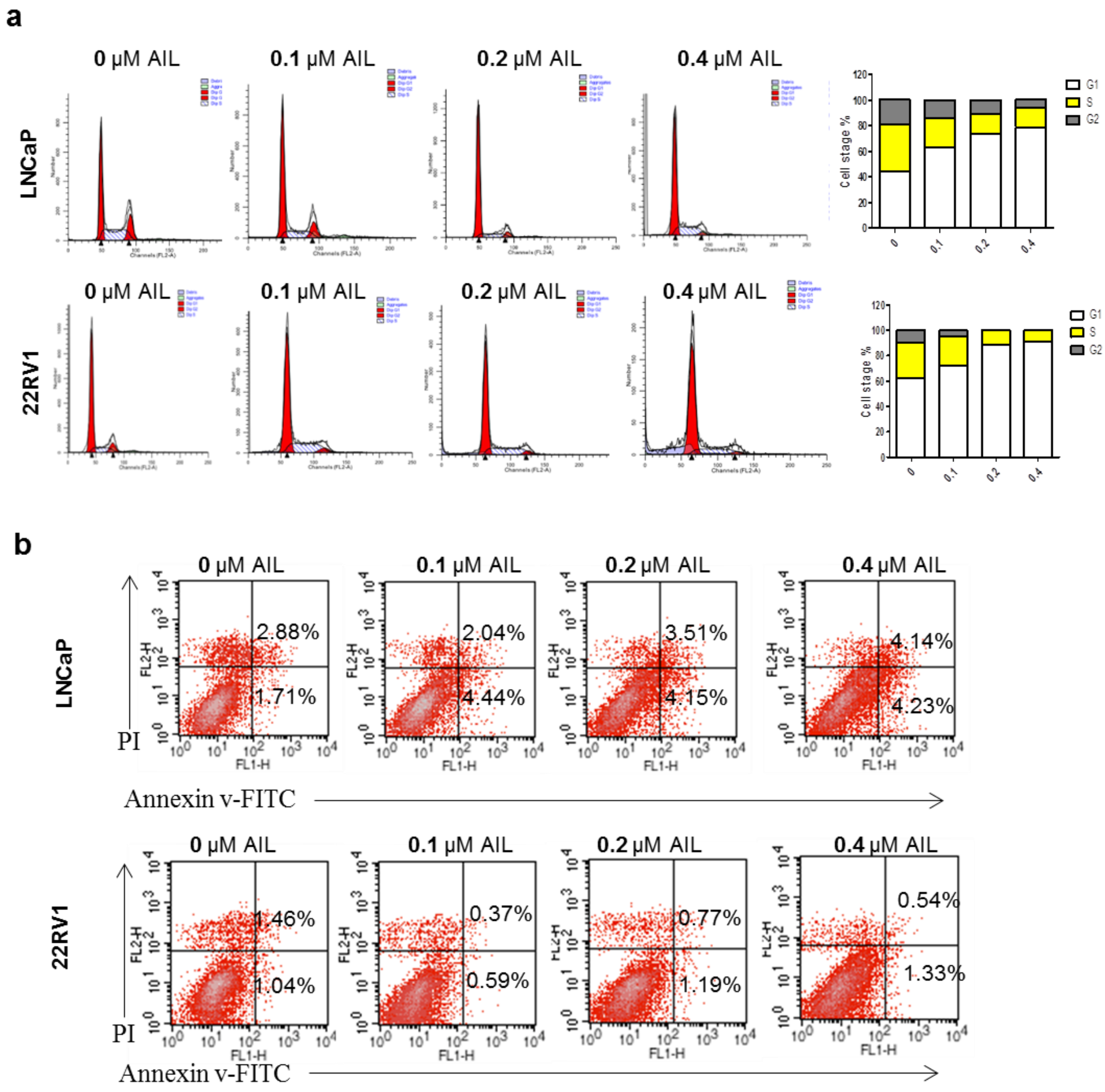

Supplementary Figure 8. AIL induced G1-phase arrest instead of apoptosis. (**a**) LNCaP and 22RV1 cells were treated with the indicated concentrations of AIL for 24 hours. Then cells were fixed with 70% ethanol and stained with PI and sent for cell cycle analysis with flow cytometry. The percentage of each phase is shown in the right panel. (**b**) LNCaP and 22RV1 cells were treated with the indicated concentrations of AIL for 24 hours. Then cells were stained with PI and Annexin V-FITC and then subjected to flow cytometry analysis of cell apoptosis.

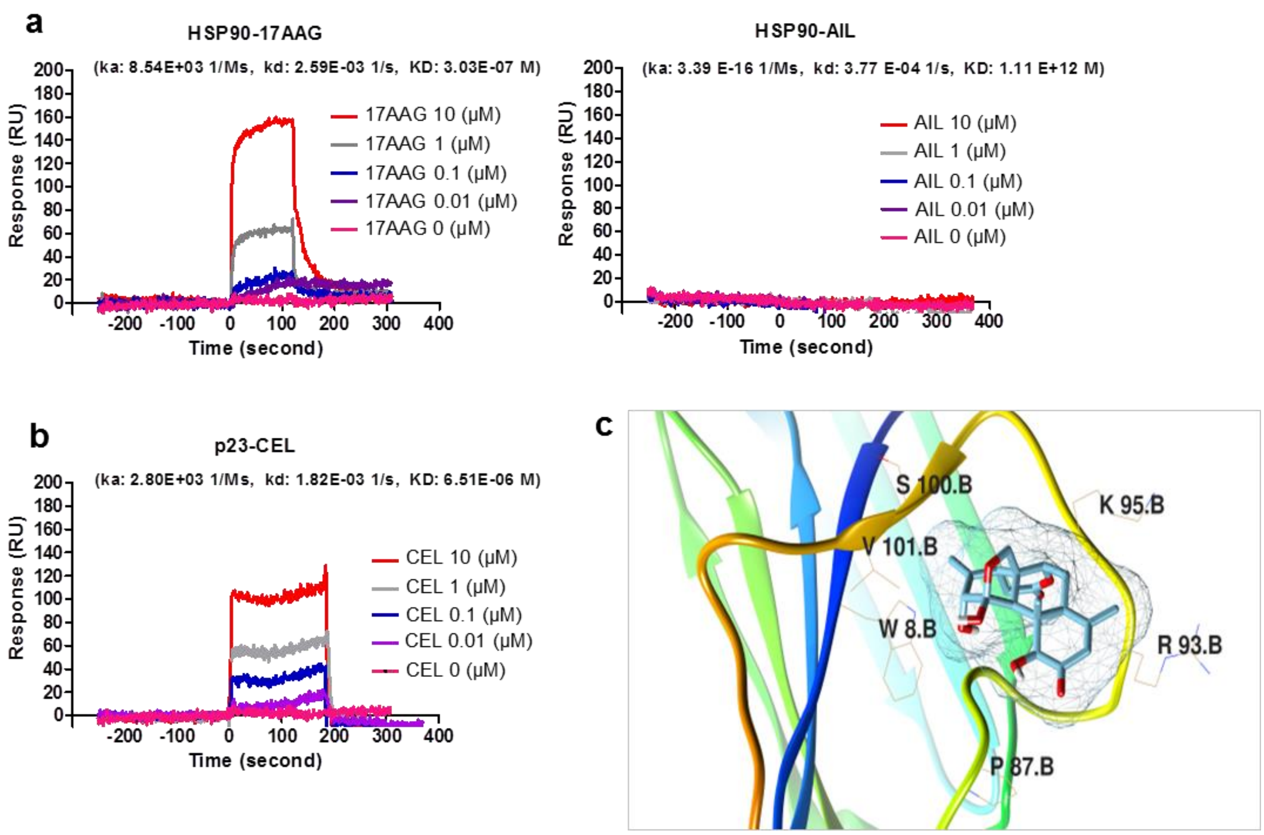

Supplementary Figure 9. AIL binds to p23 but not HSP90. (**a**) HSP90 bound to 17-AAG but not AIL *in vitro*. The interaction between HSP90α protein and AIL (Right) or 17-AAG (Left) were measured by ProteOn XPR36. (**b**) Celastrol (CEL) interacted with p23 protein *in vitro*. The interaction between p23 protein and CEL was measured by ProteOn XPR36. (**c**) Mapping AIL-binding site on p23. The docking assay was performed as described in Materials and Methods.

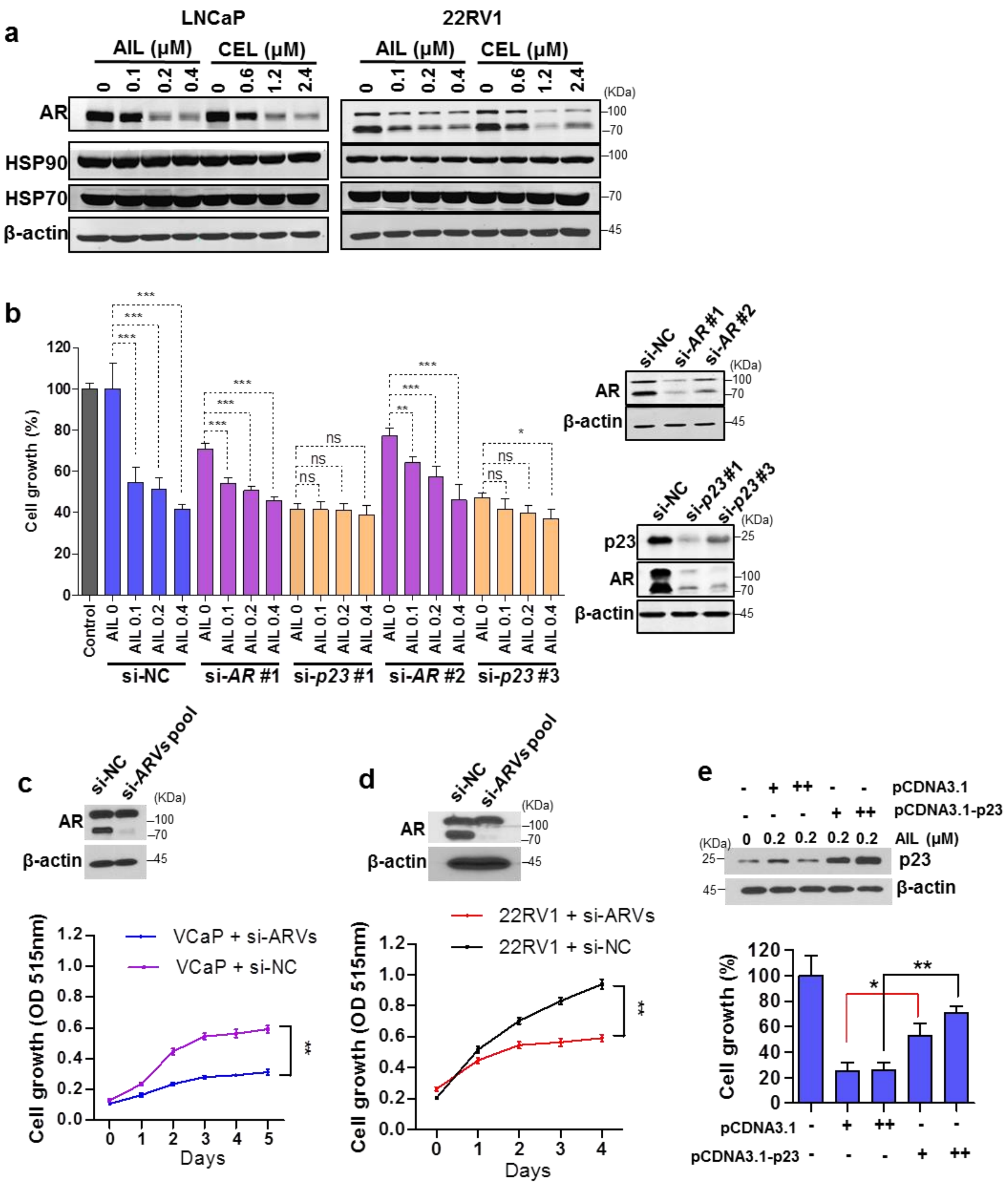

Supplementary Figure 10. AIL and a p23 inhibitor celastrol (CEL) showed the same effects on the protein level of AR, HSP90 and HSP70. (**a**) LNCaP and 22RV1 cells were treated for 12 hours with the indicated concentrations of AIL or CEL (a p23 inhibitor). Cells were lysed and AR, HSP90 and HSP70 protein levels were measured by Western blot analysis. (**b**) p23 knockdown abrogated AIL induction of cell growth arrest. 22RV1 cells with transient transfection of non-target control (si-NC), siRNA-AR or siRNA-p23 siRNAs were treated with indicated concentrations

of AIL for 72 h and cell growth was detected with the SRB assay (n = 5). Data was expressed as mean ± s.d.; Student's t-tests were performed; * $P < 0.05$, ** $P < 0.01$, *** $P < 0.001$. (**c,d**) Knockdown of AR-Vs decreased cell proliferation. VCap (c) and 22RV1 (d) cells were transfected with the AR-Vs specific siRNA pool or non-target control and the cell growth was detected with the SRB assay at the indicated times (n = 5). siRNAs were transfected every two days to maintain the knockdown efficiency. Data represent the mean ± s.d. ** $P < 0.01$, *** $P < 0.001$ by one-way ANOVA followed by Bonferroni multiple comparison test. (**e**) Overexpression of p23 rescued the AIL-mediated cell growth inhibition. 22RV1 cells were transfected with empty vector or different doses of p23 plasmid in the presence of 0.2 μM AIL for 72 hours and the cell growth was detected with the SRB assay (n = 5). Data was expressed as mean ± s.d.; Student's t-tests were performed; * $P < 0.05$, ** $P < 0.01$, *** $P < 0.001$.

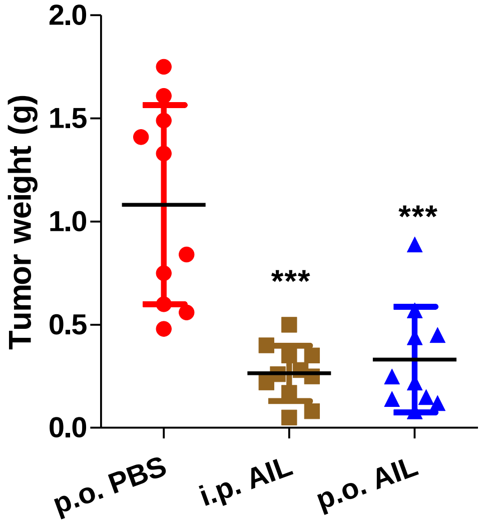

Supplementary Figure 11. The effects of *i.p.* and *p.o.* administration of AIL on the. 3×10^6 22RV1 cells were injected with 0.1 ml PBS into the right flank of nude mice. After tumor nodules were allowed to grow to a volume about 100 mm^3, the tumor bearing mice were treated with 2 mg/kg/day (*i.p.*) or 5 mg/kg/day (*p.o.*) or vehicle control (*p.o.*) for 30 days. The tumor weight was measured at the end of the experiment. Data was expressed as mean ± s.d.; Student's t-tests were performed; *** P < 0.001.

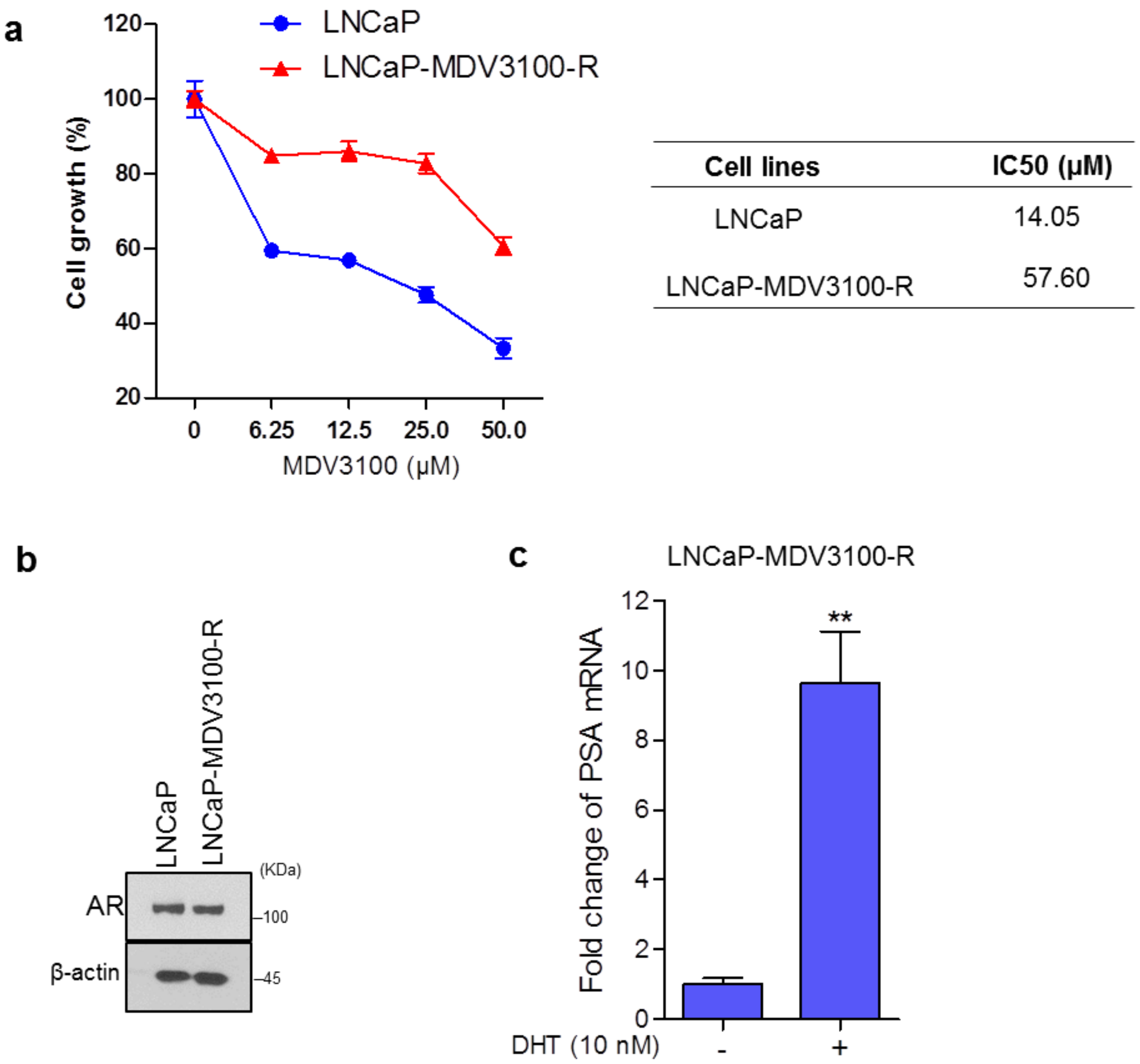

Supplementary Figure 12. Characterization of LNCaP-MDV3100-R. (**a**) LNCaP and LNCaP-MDV3100-R (resistant to MDV3100) were treated with indicated concentrations of MDV3100 for 72 hours and the cell proliferation was detected with the SRB assay (n = 5). The half maximal inhibitory concentration (IC50) was calculated using GraphPad Prism 5.0 (GraphPad, San Diego, CA). (**b**) LNCaP-MDV3100-R cell line expresses a similar AR level as LNCaP. LNCaP and LNCaP-MDV3100-R cells were lysed and AR protein levels were measured by Western blot analysis. β-actin serves as internal control. (**c**) LNCaP-MDV3100-R cells still respond to androgen. LNCaP-MDV3100-R cells were cultured in 5% c-FBS for 48 hours and treated with or without 10 nM DHT for 12 hours. DMSO was added as the control. Total RNA was extracted and quantitative-PCR was performed with the PSA and GAPDH specific primer. The *PSA* mRNA level was normalized to *GAPDH*. Data was expressed as mean ± s.d. of three independent assays; Student's t-tests were performed; ** $P < 0.01$.

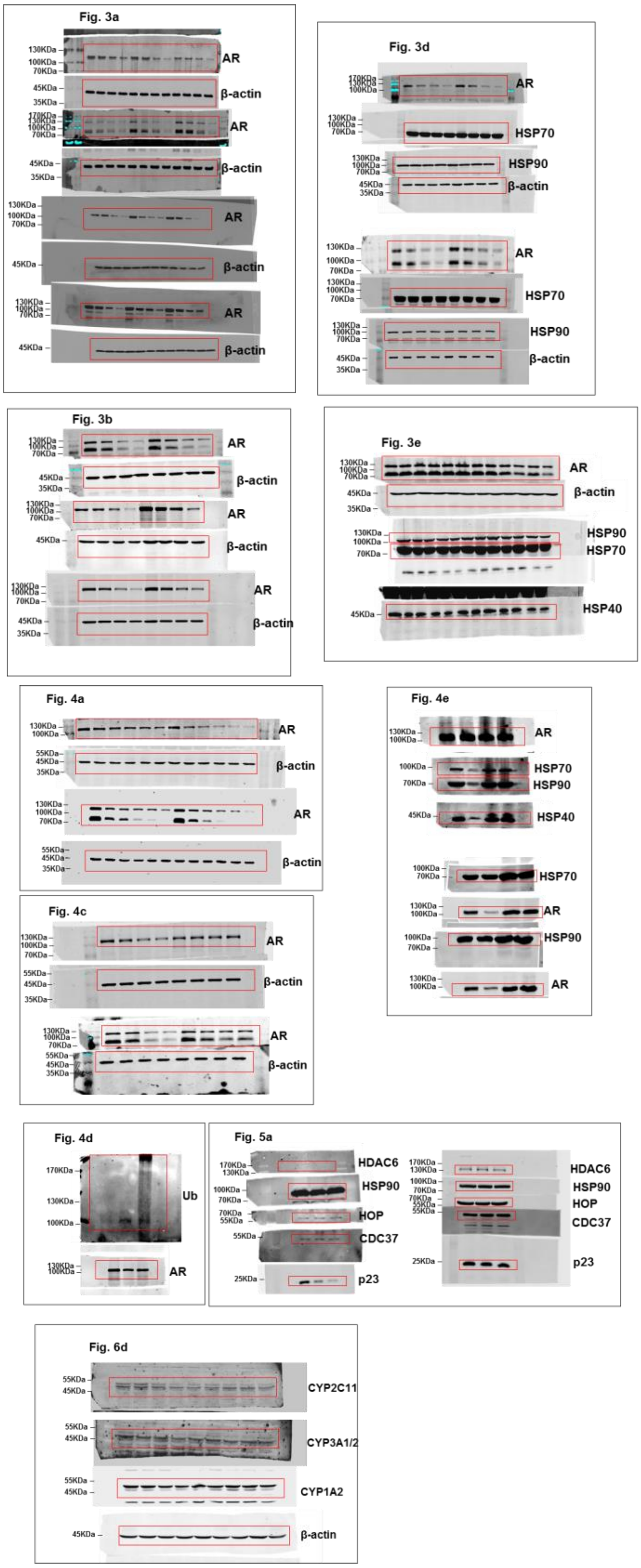

Supplementary Figure 13. Uncropped Western blots for Figure 3a, 3b, 3d， 3e， 4a, 4c, 4d, 4e, 5a and 6d.

Supplementary Table 1. Calculation of physicochemical properties of AIL

Compound	M.W	NO. of HBD	NO. of HBA	TPSA	R.B	pKa	LogP
Ailanthone (AIL)	376.4	3	7	113.29	0	12.2 ± 1.0	-0.77 ± 0.62

M.W: molecular weight; **TPSA:** Topological molecular polar surface area; **HBD:** Hydrogen Bond Donors; **HBA:** Hydrogen Bond Acceptors; **R.B:** Rotatable Bonds

Supplementary Table 2. Sequence of quantitative-PCR primers

Name of Gene	Primer (5' to 3')
AR-F	GGTGAGCAGAGTGCCCTATC
AR-R	GAAGACCTTGCAGCTTCCAC
PSA-F	CTTGTAGCCTCTCGTGGCAG
PSA-R	GACCTTCATAGCATCCGTGAG
TMPRSS2-F	CTGGTGGCTGATAGGGGATA
TMPRSS2-R	GGACAAGGGGTTAGGGAGAG
NDRG1-F	CGAGACTTTACATGGCTCTGT
NDRG1-R	TCCATGGAGGGGTACATGTA
FKBP5-F	AGAACCAAACGGAAAGGAGA
FKBP5-R	GCCACATCTCTGCAGTCAAA
SLC45A3-F	GCAGTGAGGACAGCCTGATG
SLC45A3-R	CGGAGACATCACAGGCAGAG
GAPDH-F	ACCCAGAAGACTGTGGATGG
GAPDH-R	TTCAGCTCAGGGATGACCTT
β-actin-F	GTACGCCAACACAGTGCTG
β-actin-R	CGTCATACTCCTGCTTGCTG
AR-v7-F	AAAAGAGCCGCTGAAGGGAA
AR-v7-R	CCAACCCGGAATTTTTCTCCC

Supplementary Table 3. Sequence of si-RNA

Name of Gene	si-RNA (5' to 3')
AR siRNA-1	GAAAUGAUUGCACUAUUGAUU
AR siRNA-2	CGUGCAGCCUAUUGCGAGAUU
AR-Vs pool	AR-V1: GAGGGUGUUUGGAGUCUCAUU<br>AR-V3: AAGAGCCGCUGAAGGAUUUUU<br>AR-V4: GAUGACUCUGGGAGGAUUUUU<br>AR-V7: GCAAUUGCAAGCAUCUCAAUU
p23 siRNA	AGCUUAAUUGGCUUAGUGU(dTdT)
p23 siRNA	ACACUAAGCCAAUUAAGCU(dTdT)
Nonsilencing control siRNA	AATTCTCCGAACGTGTCACGT(dTdT)

Supplementary Table 4. Screening compounds of inhibiting AR_{1-651}-induced AR activity by MMTV-luc reporter gene assay from natural compounds.

Compound Name	MMTV-luc Activity (%)	AR1-651	Compound Name	MMTV-luc Activity (%)	AR1-651
_	10.3±9.5	_	Oleanic acid (10 μM)	153.4±7.1	+
_	100.0±6.4	+	astragaloside Ⅰ (10 μM)	87.0±5.2	+
Bicalutamide (10μM)	105.8±13.6	+	Isoquercitrin (10 μM)	95.5±21.9	+
EPI-001 (25 μM)	54.2±14.6	+	Betulinic acid (10 μM)	65.2±21.5	+
Dihydromyricetin (10 μM)	89.8±8.0	+	Chrysophanol (10 μM)	88.5±16.6	+
Ergosterol (10 μM)	96.3±6.1	+	Huperzine A (10 μM)	71.5±11.0	+
Echinacoside (10 μM)	114.6±24.4	+	Isorhamnetin (10 μM)	139.1±1.8	+
Myricitrin (10 μM)	102.1±15.0	+	Gastrodin (10 μM)	70.0±3.7	+
Bilobalide (10 μM)	98.7±1.1	+	Emodin (10 μM)	69.3±15.2	+
lithospermic acid B (10 μM)	96.3±4.2	+	Tanshinone I (10 μM)	99.9±4.1	+
Pterostilbene (10 μM)	90.0±5.4	+	Pleurotus ostreatus polysaccharide (10 mg/L)	139.9±36.6	+
Ammothamnine (10 μM)	105.7±14.6	+	**Ganoderma lucidum polysaccharide (10 mg/L)**	**58.7±40.7**	+
Ginsenoside Rg1 (10 μM)	77.2±13.8	+	Onkey head mushroom (10 mg/L)	115.6±12.5	+
Sodium Danshensu (10 μM)	73.3±17.3	+	Lentinan (10 mg/L)	179.1±13.7	+
Swertiamain (10 μM)	64.7±7.5	+	Betaine (10 μM)	128.7±22.3	+
Protocatechuic aldehyde (10 μM)	62.5±11.4	+	Diosgenin (10 μM)	148.3±50.2	+
Naringin (10 μM)	69.5±14.5	+	Curcumin (10 μM)	177.6±32.2	+
L-Theanine (10 μM)	61.9±26.6	+	Kojic acid (10 μM)	179.2±4.9	+

L-Epicatechin (10 μM)	88.0±10.2	+	Arbutin (10 μM)	98.4±11.2	+
Rosmarinic acid (10 μM)	114.8±18.7	+	Herba Houttuyniae (10 μM)	85.4±0.4	+
Gallic acid (10 μM)	70.7±8.9	+	hawthorn leaves flavonoids	154.8±39.1	+
Rutin (10 μM)	91.4±13.5	+	Asiaticoside (10 μM)	150.9±57.8	+
Glycyrrhizic acid (10 μM)	111.9±5.0	+	Daidzein (10 μM)	176.8±35.5	+
Mannitol (10 μM)	83.0±31.5	+	Lycorine (10 μM)	66.0±25.1	+
Epigallocatechin gallate (10 μM)	105.4±3.1	+	Baicalin(10 μM)	112.7±17.0	+
shikimic acid (10 μM)	104.3±22.9	+	Evodine (10 μM)	69.9±39.3	+
Paeoniflorin (10 μM)	88.5±22.8	+	d-Bicuculline (10 μM)	112.1±8.0	+
**Magnolol (10 μM)**	**59.3±27.9**	+	Anemonin (10 μM)	112.4±8.1	+
Hesperidin (10 μM)	110.2±13	+	**Phlorizin (10 μM)**	**52.9±27.4**	+
Luteolin (10 μM)	82.7±4.5	+	Kaempferol (10 μM)	100.6±35.4	+
Apigenin (10 μM)	129.7±6.1	+	Tanshinone IIA (10 μM)	104.9±10.3	+
Genistein (10 μM)	156.9±23.3	+	D-Tanshinone I (10 μM)	138.3±26.4	+
Naringin (10 μM)	132.2±20.0	+	**Curcumol (10 μM)**	**43.7±11.7**	+
**Ailanthone (10 μM)**	**39.2±5.7**	+	Puerarin(10 μM)	80.1±1.8	+
Pleurotus ostreatus polysaccharide	134.2±12.8	+	Curcumol (1 μM)	86.4±7.7	+
**Andrographolide (10 μM)**	**59.5±18.7**	+	Magnolol (1 μM)	60.6±10.6	+
**Plumbagin (10 μM)**	**28.8±7.9**	+	**Ailanthone (1 μM)**	**26.0±7.2**	**+**
Quercetin (10 μM)	173.4±10.8	+	Andrographolide (1 μM)	70.0±21.6	+
glycyrrhetic acid (10 μM)	308.3±113.0	+	Plumbagin (1 μM)	128.5±20.8	+
Pogostone (10 μM)	157.2±62.8	+	Ganoderma lucidum polysaccharide (1 mg/L)	133.4±44.2	+
Salidroside (10 μM)	79.1±28.	+	Phlorizin (1 μM)	125.6±12	+

6 .2

After transient transfection of MMTV-luc, Renilla-luc and AR_{1-651} in 22RV1, the cells were treated with indicated compounds for 12 hours and the luciferase activities were measured and results were expressed as the ratio of luciferase activity. Values are presented as mean ± s.d., n = 3.

Supplementary Table 5. Screening compounds of inhibiting DHT-induced AR activity by MMTV-luc reporter gene assay from natural compounds.

Compound Name	MMTV-luc Activity (%)	DHT	Compound Name	MMTV-luc Activity (%)	DHT
_	30.5±5.6	_	Oleanic acid (10 μM)	122.4±7.5	+
_	100±10.1	+	astragaloside Ⅰ (10 μM)	117.8±5.2	+
Bicalutamide (10 μM)	45.1±1.9	+	Isoquercitrin (10 μM)	112.2±1.2	+
Bicalutamide (20 μM)	23.7±1.8	+	Betulinic acid (10 μM)	88.6±3.8	+
Dihydromyricetin (10 μM)	99.5±8.0	+	Chrysophanol (10 μM)	86.9±3.8	+
Ergosterol (10 μM)	95.7±13.7	+	Huperzine A (10 μM)	82.8±0.4	+
Echinacoside (10 μM)	104.4±9.6	+	Isorhamnetin (10 μM)	103.3±1.7	+
Myricitrin (10 μM)	124.1±0.6	+	Gastrodin (10 μM)	108.2±0.9	+
Bilobalide (10 μM)	113.5±23.9	+	Emodin (10 μM)	119.5±10.4	+
lithospermic acid B (10 μM)	115.9±27.9	+	Tanshinone I (10 μM)	60.8±3.0	+
Protocatechuic acid (10 μM)	98.9±6.4	+	Pleurotus ostreatus polysaccharide (10 mg/L)	118.7±8.2	+
Ammothamnine (10 μM)	119.1±20.9	+	Ganoderma lucidum polysaccharide (10 mg/L)	120.9±7.9	+
Ginsenoside Rg1 (10 μM)	119.8±28.2	+	Onkey head mushroom (10 mg/L)	120.7±6.0	+
Sodium Danshensu (10 μM)	93.0±14.7	+	Lentinan (10 mg/L)	86.1±2.5	+
Swertiamain (10 μM)	80.9±8.5	+	Betaine (10 μM)	105.1±1.9	+
Protocatechuic aldehyde (10 μM)	83.1±0.1	+	Diosgenin (10 μM)	109.7±4.9	+
Naringin (10 μM)	88.9±1.3	+	Curcumin (10 μM)	69.7±5.6	+
L-Theanine (10 μM)	89.4±9.5	+	Kojic acid (10 μM)	134.7±11.3	+
L-Epicatechin (10 μM)	86.6±4.6	+	Arbutin (10 μM)	135.5±3.9	+
Rosmarinic acid (10 μM)	90.4±3.4	+	Herba Houttuyniae (10 μM)	110..0±1.6	+
Gallic acid (10 μM)	98.2±3.5	+	hawthorn leaves flavonoids	100.2±3.9	+
Rutin (10 μM)	95.7±11.1	+	Asiaticoside (10 μM)	101.1±8.5	+
Glycyrrhizic acid (10 μM)	96.5±0.4	+	Daidzein (10 μM)	130.3±23.8	+

Mannitol (10 μM)	103.8±4.1	+	Lycorine (10 μM)	39.5±4.4	+
Epigallocatechin gallate (10 μM)	91.4±3.4	+	glycyrrhetic acid (10 μM)	101.3±11.9	+
shikimic acid (10 μM)	89.1±2.1	+	Evodine (10 μM)	84.4±6.3	+
Paeoniflorin (10 μM)	81.6±4.0	+	d-Bicuculline (10 μM)	75.0±11.5	+
Magnolol (10 μM)	92.3±7.3	+	Anemonin (10 μM)	79.2±11.2	+
Hesperidin (10 μM)	85.5±23.9	+	Paclitaxel (10 μM)	73.3±8.4	+
Luteolin (10 μM)	180.7±3.9	+	Kaempferol (10 μM)	131.9±3.9	+
Apigenin (10 μM)	135.8±8.0	+	Tanshinone IIA (10 μM)	150.4±23.4	+
Genistein (10 μM)	118.1±21.1	+	Phlorizin (10 μM)	101.3±25.6	+
Naringin (10 μM)	104.2±8.6	+	Curcumol (10 μM)	90.0±9.2	+
Ailanthone (10 μM)	38.4±3.2	+	Andrographolide (10 μM)	96.1±7.8	+
Curcumol (10 μM)	90.0±9.2	+			
Andrographolide (10 μM)	96.1±7.8	+	Bicalutamide (0.5 μM)	93.0±6.4	+
Plumbagin (10 μM)	32.1±3.6	+	Ailanthone (0.5 μM)	24.4±2.1	+
Quercetin (10 μM)	114.2±9.9	+	Tanshinone I (0.5 μM)	62.6±5.6	+
glycyrrhetic acid (10 μM)	101.3±11.9	+	Curcumin (0.5 μM)	92.6±3.0	+
Pogostone (10 μM)	92.3±13.3	+	Lycorine (0.5 μM)	107.8±17.8	+
Salidroside (10 μM)	88.8±1.3	+	Plumbagin (0.5 μM)	88.4.±2.8	+

After transient transfection of MMTV-luc and Renilla-luc in 22RV1, the MMTV-luc activities were stimulated by androgen DHT. Then cells were treated with indicated compounds for 12 hours and the luciferase activities were measured and results were expressed as the ratio of luciferase activity. Values are presented as mean ± s.d., n = 3.

Supplementary Methods

AR siRNA and p23 siRNA assay

22RV1 cells were seeded in 6-well plates and transfected with *AR*-siRNA and *p23*-siRNA (synthesized by Biotend, Shanghai, China) the next day. Transfection was performed using HyliMax REAGENT according to the manufacturer's recommendations (Dojindo Laboratories, Japan). The effect of siRNA on AR and p23 silencing was examined by Western blot 72 hours after transfection. After transfection for 16 hours, cells were treated with various concentrations of AIL for 72 hours. The gene-specific siRNAs are listed in Supplementary Table S3.

Cell cycle analysis

After treatment with different concentrations of AIL, cells were trypsinized, washed with PBS and fixed with cold 70% ethanol at 4°C overnight. Cells were then washed with PBS, treated with 50 µg/ml RNase at 37°C for 20 min and stained with 20 µg/ml propidium iodide solution for 15 min at room temperature in the dark. The stained cells were analyzed using BD LSRII flow cytometry (BD Biosciences).

Cell apoptosis analysis

After treatment with different concentrations of AIL, cells were trypsinized, washed with PBS and stained with 20 µg/ml propidium iodide (PI) solution and Annexin V-FITC for 15 min at room temperature in the dark. The stained cells were analyzed using BD LSRII flow cytometry (BD Biosciences).

RNA-seq

LNCaP cells were transfected with $AR_{1\text{-}651}$ or empty vector as control for 24 hours and cells were treated with or without AIL for the next 12 hours. Total RNAs were isolated from cells and RNA was isolated using RNeasy Plus Mini Kit (Qiagen). High quality (Agilent Bioanalyzer RIN >7.0) total RNAs were employed for the preparation of sequencing libraries using Illumina TruSeq Stranded Total RNA/Ribo-Zero Sample Prep Kit. A total of 500–1,000 ng of riboRNA-depleted total RNA was fragmented by RNase III treatment at 37°C for 10–18 min and RNase III was inactivated at 65°C for 10 min. Size selection (50 to 150 bp fragments) was performed using the FlashPAGE denaturing PAGE-fractionator (Life Technologies) prior to ethanol precipitation overnight. The resulting RNA was directionally ligated, reverse-transcribed and RNase H treated. Samples were sequenced using the Illumina HiSeq2000 platform at The Beijing Genomics Institute (BGI) in Wuhan, China. Genome-wide coverage signals were represented in BigWig format to facilitate convenient visualization using the UCSC genome browser. Gene expression was measured using RPKM (Reads Per Kilo-base exon per Million mapped reads) as described previously[1].

Gene Ontology (GO) analysis

Using DAVID Bioinformatics Resources v6.7, a web-based functional annotation tool

for data analysis (http://david.abcc.ncifcrf.gov/home.jsp), we performed gene ontology (GO) analysis for biological processes (GOTERM_BP_FAT) of the $AR_{1\text{-}651}$-induced genes inhibited by AIL treatment.

ProteOn XPR36 protein interaction array (Biacore assay)

To measurement of binding affinities between AIL and p23 as well as HSP90, p23 (Abcam, cat. no. ab75542) and HSP90α (BPS, cat. no. 50290) were dissolved in PBS and immobilized onto separate erect channels of the sensor chip by general amine coupling. p23 was immobilized to around 23300 RUs and HSP90 was 43100 RUs. After baselines were stable, AIL was dissolved in PBS-T buffer flowing through the chip horizontally. 17-AAG (binding to HSP90) and celastrol (binding to p23) were performed as positive controls. Data were analyzed with ProteOn manager™ software using the Langmuir model (A+B ↔ AB) for kinetic data fitting.

Pharmacokinetic study of ailanthone in rats

Sprague-Dawley rats (purchased from Shanghai SLAC laboratory animal Co. Ltd., Shanghai, China) were fasted for 12 h with free access to water prior to the pharmacokinetic study. The rats were randomized into two groups for oral administration (5mg/kg) and intravenous injection (1mg/kg). Blood samples were collected from the orbital plexus into heparinized centrifuge tubes at fifteen different time points between 5 min to 36 h for the oral administration group and nine time points between 5 min to 6 h for the intravenous one. The pharmacokinetic parameters were calculated by WinNonlin software version 5.2.1 based on noncompartmental analysis. Oral bioavailability was calculated as $F\ (\%) = AUC_{0\text{-}\infty}(p.o.)\ /\ AUC_{0\text{-}\infty}(i.v.) \times Dose\ (i.v.)\ /\ Dose\ (p.o.)$.

Mapping the AIL-binding site on p23

In order to identify how AIL bound to p23, we applied the computational docking modeling process based on the reported literature[2, 3, 4]. The docking process was shown in the AIL-p23 Docking Studies. Though analysis of docking mode, these results showed that the phenyl of residue Trp-8, as well as the alkyl parts of Ser-100, Val-100, Lys-95 and Arg-93 provided hydrophobic interactions with AIL (Figure 5C and Supplementary Figure 9C). The indol N-H of Trp-8, the carboxyl of Pro-87 and the NH2 of Arg-93 provided the hydrophilic interactions with AIL (Figure 5C and Supplementary Figure 9C). This binding mode provides the possibility that AIL could insert into the pocket on the surface of p23 which was formed by Ser-100, Val-100, Lys-95, Arg-93 and Trp-8.

Analysis of physicochemical properties of AIL

The various physicochemical properties were calculated for AIL using the ACD/I-Lab website (https://ilab.acdlabs.com/iLab2/index.php). The natural compound AIL had favorable results in physicochemical properties of partition coefficient of LogP (ACD/Labs) and surface area calculations of TPSA. According to molecular weight calculations, AIL was below 400 Da with a high oral absorption value. AIL had

favorable physicochemical properties as the pKa was 12.2 ± 1.0. The detailed data was listed in the Supplementary Table 1.

Supplementary References

1. Mortazavi A, Williams BA, McCue K, Schaeffer L, Wold B. Mapping and quantifying mammalian transcriptomes by RNA-Seq. *Nature methods* **5**, 621-628 (2008).

2. Patwardhan CA, Fauq A, Peterson LB, Miller C, Blagg BS, Chadli A. Gedunin inactivates the co-chaperone p23 protein causing cancer cell death by apoptosis. *The Journal of biological chemistry* **288**, 7313-7325 (2013).

3. Martinez-Yamout MA, Venkitakrishnan RP, Preece NE, Kroon G, Wright PE, Dyson HJ. Localization of sites of interaction between p23 and Hsp90 in solution. *The Journal of biological chemistry* **281**, 14457-14464 (2006).

4. Chadli A*, et al.* Celastrol inhibits Hsp90 chaperoning of steroid receptors by inducing fibrillization of the Co-chaperone p23. *The Journal of biological chemistry* **285**, 4224-4231 (2010).
